# Optimizing Athlete Travel for Performance: A Scientific Blueprint for Athletes, Coaches, and Sports Medicine Staff

**DOI:** 10.1007/s40279-026-02455-y

**Published:** 2026-05-26

**Authors:** Nicolas Hatamiya, Kristen E. Holmes, Gregory J. Grosicki, Jeremy Swisher, Calvin Duffaut, Jeremy Vail, Heather Logan-Sprenger, Brian Donohoe, Finnbarr Fielding, Christopher J. Chapman, Josh Leota, Elise R. Facer-Childs, Joshua T. Goldman

**Affiliations:** 1 Department of Orthopaedic Surgery, Department of Family and Community Medicine, University of California, 1500 Owens Street, San Francisco, CA 94158 USA; 2 Department of Performance Science, WHOOP, Inc., Boston, MA USA; 3 Division of Sports Medicine, Department of Family Medicine, University of California, Los Angeles, CA USA; 4 Department of Intercollegiate Athletics, University of California, Los Angeles, CA USA; 5 Faculty of Health Sciences, Ontario Tech University, Oshawa, Canada; 6 Sleep and Circadian Rhythms Research Program, School of Psychological Sciences, Faculty of Medicine, Nursing and Health Sciences, Monash University, Melbourne, VIC Australia; 7 Division of Sleep and Circadian Disorders, Department of Medicine, Brigham and Women’s Hospital, Boston, MA USA; 8 Department of Research, Algorithms, and Data Science, WHOOP, Inc., Boston, MA USA

## Abstract

Travel is an integral component of modern sports, with athletes frequently crossing timezones for competition. This travel introduces challenges that can impact both recovery and athletic performance. As more athletes and teams travel for competition, it is increasingly important to understand ways to mitigate common travel-related issues such as jet lag, travel fatigue, and sleep disturbances. Specific strategies to adapt to new timezones including managing light exposure, ensuring proper hydration and fueling, determining appropriate travel times, utilizing supplements and maintaining sleep consistency should be addressed. Additional considerations include the potential impact of other environmental factors, such as adapting to heat or altitude, when combined with traveling. In this narrative review, we focus on long-haul travel, where circadian misalignment and jet lag are most pronounced, and provide a scientific blueprint of how to minimize the impacts of travel on athletes with the goal of helping athletes, coaches, and sports medicine staff to develop a practical framework to enhance recovery and athletic performance amidst travel-related obstacles.

## Key Points

**Table:** 

As athlete travel becomes more prevalent, particularly long-haul travel crossing multiple timezones, the impacts of travel on athlete performance should be considered as they pertain to jet lag, travel-related fatigue, fueling, gastrointestinal disturbances, environmental conditions, illness, and soft-tissue injury.
Disruptions of sleep and normal circadian rhythms can degrade mental functioning, mood, reaction time, and decision making which can affect physical performance metrics.
The current body of literature provides some recommendations for ways athletes can mitigate these impacts across hydration, fueling, supplementation, optimal timing of travel around competition, circadian alignment, sleep behavior, and injury/illness mitigation.

## Introduction

**Fig. 1:**
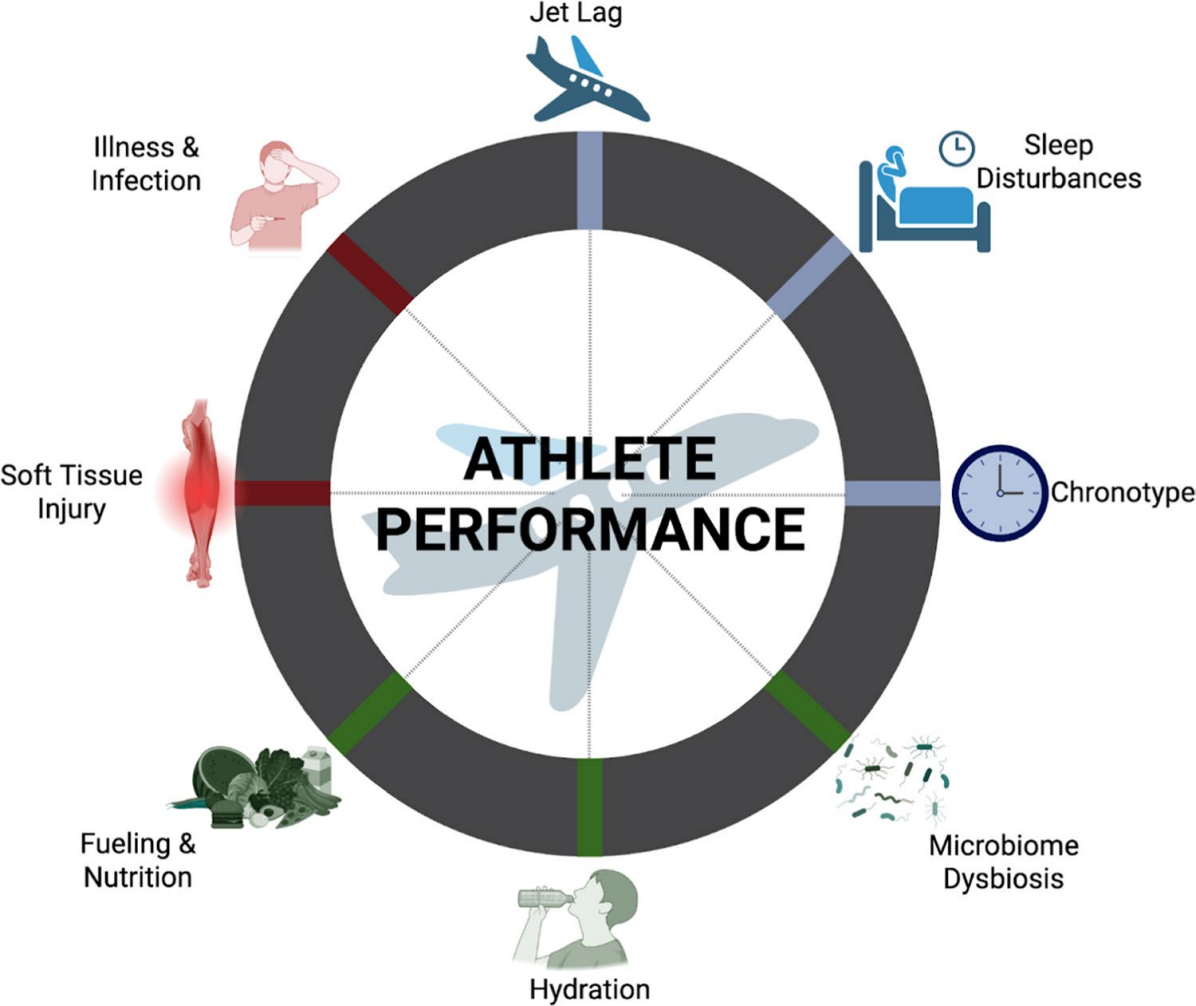
Impact of travel across timezones on athletic performance (image created using BioRender)

In the current era of sport, elite athletes and teams face unprecedented travel demands, routinely flying long distances for competitions ranging from domestic league games to international competitions. Travel has become an essential component of sport, yet it poses well-documented challenges to athlete health and performance [1]. Long-haul, rapid transit across multiple timezones presents athletes with two distinct challenges: jet lag and travel fatigue [2, 3]. Jet lag is the circadian misalignment that occurs when the body’s internal clock is ‘out of sync’ with local time at the destination, whereas travel fatigue is the general physical and mental exhaustion from the travel process itself (e.g., sleep deprivation, dehydration, prolonged sitting, irregular schedules, and stress). While travel fatigue can occur with any form of travel, jet lag occurs when crossing multiple timezones. The combined impact of both jet lag and travel fatigue is particularly pronounced following long-haul, trans-meridian flights. The sports medicine community has grown increasingly concerned with these long-haul travel-related stressors, recognizing that, if left unmitigated, they may impair multiple aspects related to an athlete’s physiological recovery, cognitive function, general health, and competitive capacity in the days following travel (Fig. 1) [4, 5].

The circadian disruption caused by jet lag from long-haul travel warrants particular consideration. For athletes, this circadian misalignment can translate into suboptimal performances – for example, through decreased reaction time, impaired decision making, and altered exercise capacity at critical moments [1, 2]. Athletic performance normally oscillates on a roughly 24-h cycle, peaking when circadian rhythms cue optimal muscle strength, coordination, and mental acuity in the late afternoon or early evening for most sports [3, 6]. Peak performance timing varies by athletic event, and for endurance sports such as marathon running, a morning start is advantageous, likely due to cooler ambient temperatures [7]. Traveling across timezones can blunt or shift these natural peaks. Although empirical studies show variable effects on competition outcomes, likely due to individual and situational differences, the consensus is that significant travel without proper adjustment is a risk factor for performance decrement [4]. Notably, teams with a circadian “advantage” (i.e., physiologic clocks aligned to the competition time) have been shown to win more often, underscoring the subtle yet impactful role of circadian timing on sporting success [8]. In addition to timezone shifts, the general fatigue of long-haul travel may itself hinder recovery by disturbing sleep patterns even without circadian misalignment, though direct evidence for this independent effect remains limited. Thus, both transmeridian (jet lag) and non-timezone travel fatigue should be addressed in high-performance settings.

Beyond circadian and fatigue-related performance effects, extensive travel may also compromise athletes’ health and well-being if not managed appropriately. Immune function can be suppressed by the stress of travel and disrupted sleep, which in turn may increase susceptibility to illness during or after trips [5]. Indeed, a landmark study of elite athletes found that traveling to international destinations with > 5 h time differences was associated with a two- to threefold higher risk of illness, such as upper respiratory infections, in the week following travel [9]. Frequent travel also often means exposure to crowded airports, planes, and new environments, creating opportunities for infectious diseases to spread through teams. Moreover, accumulated fatigue from travel might heighten injury risk indirectly; for example, chronic sleep deprivation is known to impair psychomotor function and has been linked to higher injury rates in athletes [5]. These health risks highlight that optimizing travel is not merely about maintaining performance, but also about protecting athletes from preventable illness and injury. Sports organizations now consider travel a key component of athlete load management and wellness monitoring [10].

Fortunately, a growing body of literature in circadian science and sports medicine offers guidance on how to mitigate long-haul travel’s negative impacts on athletes [1, 10, 11]. Strategies such as gradually shifting sleep schedules before departure, strategic light exposure (and avoidance) to facilitate circadian re-entrainment, use of melatonin or other physiologic sleep aids, and careful planning of training timing and nutritional intake around travel have all been proposed to help athletes adapt more quickly [1, 11]. Practical travel logistics – from flight scheduling and layover management to hydration and hygiene during transit – can also make a meaningful difference to the health and well-being of athletes and are increasingly emphasized by performance staff. Despite these recommendations, high-quality evidence in athlete populations remains limited. A recent systematic review noted a lack of controlled trials on travel fatigue interventions and only low-quality evidence guiding jet-lag management in sport [10]. This gap underscores the need for continued research and consensus-building, even as teams must act on the best information currently available.

In this narrative review, we synthesize the latest scientific evidence and consensus best practices into a pragmatic blueprint for managing travel in sport. Our aim is to provide sports medicine providers, athletes, and team administrators with an evidence-informed framework to enhance performance and recovery while minimizing illness and injury during periods of travel. By integrating principles of circadian rhythm optimization, sleep science, and clinical sports medicine, this review offers actionable strategies to help athletic populations navigate the rigors of travel. Ultimately, proactive travel management can be a competitive edge and a safeguard for athlete health – an increasingly important priority as sports continue to globalize and the travel demands on athletes grow. The following sections detail the physiological basis of travel-related issues and outline practical approaches to attenuate these challenges, forming a scientific blueprint to support athletes on the move.

### Literature Search and Article Selection

This narrative review was informed by a targeted, non-systematic search of the sports medicine literature using PubMed/MEDLINE, Embase, and Google Scholar through August 2025. Search terms included combinations of keywords and medical subject headings related to the topic, athlete populations, and performance outcomes. Articles were selected based on clinical relevance and methodological quality, with priority given to randomized controlled trials, systematic reviews and meta-analyses, consensus statements, and high-quality observational studies. Studies were excluded if they were not published in English.

## Minimizing Travel-Related Pathology

### Jet Lag and Travel-Related Fatigue

Circadian rhythms regulate a broad range of physiological processes essential to athletic performance and recovery, including hormone secretion, core body temperature, cardiovascular efficiency, and the sleep–wake cycle [12–14]. These rhythms are synchronized to the 24-h day primarily by light–dark exposure, but also by behavioral cues such as exercise, eating, and social interaction [15–17]. When athletes travel across timezones, endogenous circadian clocks become misaligned with the external environment, a phenomenon commonly referred to as jet lag. This transient circadian disruption can desynchronize internal physiological rhythms and impair regulatory processes, leading to disrupted sleep, reduced alertness, hormonal imbalance, gastrointestinal discomfort, and performance decrements [18–20]. Re-entrainment to the new timezone typically occurs at a rate of approximately 1 h per day, meaning full adaptation after long-haul travel can take several days. It is also important to consider the directionality of travel [21–23]. Notably, eastward flights tend to cause worse jet lag than westward flights, since phase advances (required when flying east) are harder for the circadian system than phase delays (flying west) [24]. In practical terms, “losing” hours going east is more disruptive than “gaining” hours going west, as the body’s clock adjusts more easily by delaying than by advancing.

In contrast, travel-related fatigue refers to the cumulative physical and psychological strain associated with the travel process itself and is independent of timezone changes. Travel-related fatigue can arise from prolonged immobility, disrupted routines, dehydration, uncomfortable seating arrangements, and psychological stress [10, 20, 25]. Unlike jet lag, travel fatigue can occur after any lengthy trip, including north–south travel, and is often compounded by tight competition schedules or frequent back-to-back travel. Symptoms may include general fatigue, reduced motivation, muscle stiffness, impaired concentration, and increased perceived exertion [11, 26]. Both jet lag and travel-related fatigue are important to recognize since they can lead to daytime fatigue, poor concentration, and sleep disruption, increasing injury and illness risks [11, 20].

### Sleep Disturbances

Adequate sleep is essential for athletic performance, recovery, and cognitive function, yet travel-related sleep disruptions are common [27]. Studies show elite athletes have reduced total sleep time and sleep efficiency for ~ 2 days after long-haul flights, commonly defined as flights lasting longer than 6 h or crossing three timezones [28, 29]. Sleep diaries report worse sleep quality and increased pre-bed fatigue post-travel. Circadian misalignment can also impair athletes’ cognitive and physical performance. Disruptions of sleep and normal circadian rhythms degrade mental functioning, mood, reaction time, and decision-making, and can diminish various physical performance metrics [2]. Therefore, allowing one recovery day per timezone before competition is recommended [20]. In unfamiliar hotels, excess light or noise can disrupt sleep, and in-flight conditions (noise, cabin pressure, odd meal timing, cramped seating) further degrade sleep quality. Additional recommendations to reduce the impact of sleep disturbance are addressed throughout this paper as well as practical suggestions to help mitigate this (Table 1).

**Table 1:** Practical sleep hygiene and travel tips

Athletes and staff should enforce good sleep habits and behaviors in transit and on location
Create a dark, quiet, and cool sleep environment – use eye masks, earplugs, white-noise apps, and keep room temperature ~ 18 °C in males and ~ 19–20 °C in females [220]
Pack familiar items (pillow, blanket) to improve comfort in hotels
Limit blue light screen exposure and avoid large meals within ~ 2–3 h of bedtime (especially high-fat, spicy, or high-fiber foods) to facilitate sleep onset
Gradually shift bed and wake times toward the destination time in the days before departure (“phase shifting”) to pre-empt jet lag [24]. Upon arrival, immediately switch to local schedules for meals and sleep
Light exercise outdoors can help fight daytime grogginess while reinforcing the new timezone. Coaches should build flexibility into training plans post-travel – lighter sessions at first and extra recovery opportunities (e.g., post-flight naps, massage)
Combining circadian-aligned behaviors (light/dark exposure and melatonin timing) with diligent sleep habits and behaviors, and logistical planning can substantially mitigate travel-related sleep disturbances, helping athletes stay healthy and perform at their best across timezones [20]

### Illness and Infection

Travel can also have an impact on the natural risk of illness. Traveling to foreign destinations has been associated with a two- to threefold increase in incidence of illness [30]. The most common causes of infection include traveler’s diarrhea, respiratory tract infections, vector-borne diseases (such as malaria and Zika) and skin infections [9]. Strategies to reduce the risk of traveler’s diarrhea and other food-borne illnesses include drinking safe, potable water, leveraging local knowledge when selecting restaurants, avoiding buffet-style catering, washing and peeling fruits and vegetables, and practicing good personal hygiene such as keeping hands away from the face and undertaking frequent hand washing with soap and water for 20 s, and use of sanitizer with at least 60% alcohol content [9]. To help prevent the spread of illness, appropriate coughing and sneezing techniques can also be encouraged along with use of facial coverings by those with illness. Face coverings may also be considered for those who are not ill as a way to minimize exposure to pathogens in crowded areas. Athletes should be up to date on routine vaccinations such as tetanus, diphtheria, pertussis, influenza, hepatitis A, hepatitis B, measles, mumps, and varicella along with travel-specific immunizations to have maximal defense against infection [9]. To help prevent vector-borne disease, malaria chemoprophylaxis should be prescribed for travel to endemic regions. Also, it is recommended to use personal protective measures such as insect repellents and permethrin-treated clothing, and minimize skin exposure [31].

Several lifestyle considerations can further aid in the minimization of illness, including hydration and nutrition, and these are further explored in this review. Supplements including zinc along with vitamin C and D have been found to help support immune function [32]. Also, use of probiotics may decrease upper respiratory tract infection symptom severity, which is further discussed in this review [33]. Getting adequate sleep can boost the immune system and decrease the infection risks seen with travel, while finding ways to help manage stress can keep the immune system strong [34]. Avoiding smoking tobacco is beneficial, as its use has been shown to increase vulnerability to infection [35].

### Soft Tissue Pathology

Soft tissue pathology/injury has the potential to cause both short- and longer-term disruption to an athlete’s competitive season, with a high risk of recurrence, cited at 1.5 to over 4.8 times the relative risk [36]. While travel specifically is poorly linked in the literature, there is moderate support for a causal link between sleep and soft tissue injury occurrence specifically in the military population [37]. Adolescent athletes may be at a 1.7 times increased risk for a sports-related injury when comparing an average night’s sleep of < 8 h versus ≥ 8 h [38]. It has also been suggested that acute short sleep duration (< 6 h the night before) may be correlated with sports-related injuries [39].

While there exists no practical way to avoid travel in sport, and no clear intervention related to soft tissue injury reduction and travel, preventative measures would suggest optimization of sleep, with a potential to decrease the sleep risk factor by advocating for earlier arrivals for away matches when possible. Additionally, attempts should be made to control other modifiable risk factors anecdotally related to soft tissue injury, such as hydration and micro/macronutrient supplementation.

### Hydration

Long-haul air travel presents a meaningful challenge to fluid balance in athletes due to a combination of low cabin humidity (< 20%), prolonged immobility, altered thirst perception, and reduced voluntary fluid intake. Aircraft cabins are typically pressurized to the equivalent of ~ 2400 m altitude, which increases respiratory water loss and contributes to subclinical hypohydration during prolonged flights [40, 41]. Experimental simulations of long-haul flights (~ 10 h) have demonstrated reductions in plasma volume of approximately 6–9%, accompanied by peripheral fluid redistribution and lower-limb edema, rather than consistent whole-body mass loss [40, 41].

The performance consequences of hypohydration are well established, with losses ≥ 2% of body mass shown to impair endurance capacity, thermoregulation, and cardiovascular stability, and increase perceived exertion during exercise [42]. More severe hypohydration (~ 3% body mass loss) has been associated with impairments in muscle strength, power, anaerobic capacity, and cognitive function [43, 44]. However, there is currently limited evidence demonstrating that athletes routinely experience this magnitude of body mass loss as a direct result of air travel alone, even following long-haul flights. Instead, the primary hydration-related concern following travel appears to be plasma volume contraction and delayed rehydration, which may exacerbate cardiovascular strain and thermoregulatory stress once training or competition resumes.

To mitigate travel-related fluid disturbances, athletes should begin long-haul travel in a euhydrated state and consume fluids regularly throughout the flight. General recommendations suggest targeting approximately 15–20 mL·h⁻^1^ of fluid intake during flight, with consideration for individual tolerance, flight duration, and environmental conditions at the destination [45]. Electrolyte-containing beverages may be more effective than water alone for preserving plasma volume and promoting fluid retention by attenuating urine output [40].

Alcohol intake should be avoided due to its diuretic properties and negative effects on sleep and recovery [46]. While moderate habitual caffeine intake does not appear to exacerbate dehydration, caffeine use should be strategically timed, particularly following long-haul travel where sleep and circadian realignment are priorities [47, 48]. Caffeine consumption should be minimized in the biological evening or in the hours preceding planned sleep to avoid impairing sleep onset and prolonging circadian misalignment. Upon arrival, monitoring hydration status using urine color, body mass trends, and subjective thirst can assist athletes and staff in guiding individualized rehydration strategies.

### Microbiome Changes

Gastrointestinal (GI) distress is a well-documented consequence of travel that can undermine training and competitive performance [49]. Among international travelers, more than half report new GI symptoms during or shortly after travel [50]. Among athletes, traveler’s diarrhea is typically linked to ingestion of contaminated food or water, and while often self-limited, may require medical treatment [51]. Because antibiotic use can carry side effects and performance-relevant risks, prevention and symptom mitigation remain key considerations for traveling athletes [49, 52].

Travel-related GI symptoms have been associated with alterations in the gut microbiome, which is a complex community of micro-organisms essential for digestion, immune function, and intestinal barrier integrity [53]. In athletes, the microbiome contributes to exercise metabolism and immune defense, whereas microbial dysbiosis has been associated with impaired digestive and immune function [54–58]. In addition to the documented acute and chronic effects of exercise on the gut microbiota and its metabolic byproducts, emerging evidence suggests that greater microbiome stability may be associated with more favorable training adaptations [59–63]. International travel has been shown to reduce microbial diversity, alter taxonomic composition, and increase the prevalence of potentially pathogenic and antibiotic-resistance-associated taxa, even in the absence of overt illness [63–65]. These changes may be further influenced by travel-related stressors, including dietary changes and circadian misalignment, which have been independently linked to microbiome instability and inflammatory responses [66–68].

Interest has therefore grown in interventions aiming at mitigating travel-related GI symptoms, including probiotic supplementation [69]. Evidence in athletes suggests that probiotics may reduce the severity of upper respiratory tract infection symptoms and attenuate GI distress during endurance events; however, findings related to immune and inflammatory markers are inconsistent, and few studies have examined travel-specific outcomes [33, 70, 71]. Effects appear highly dependent on probiotic strain, dose, and host factors. As such, probiotic use may be considered on an individual basis for athletes with a history of travel-related GI symptoms, but current evidence does not support routine or universal supplementation for travel-related microbiome protection.

## Optimizing Travel and Timing for Competition

### Traveling West to East Versus East to West

The human circadian rhythm typically runs slightly longer than 24 h, making it easier to adjust to lengthened days, as experienced during westward travel, since delaying sleep and activity schedules aligns more naturally with our internal clocks. However, even with this advantage, crossing multiple timezones westward can still lead to circadian misalignment. The symptoms of jet lag might be uncomfortable and disruptive during and after a single short-duration trip involving one or two timezones, but even these small disturbances of circadian rhythms can have effects on athletic performance by impairing physical and mental reaction time [72, 73].

Both eastward and westward travel can negatively affect athletic performance, with the magnitude of disruption likely increasing as a function of the number of timezones crossed. Eastward travel generally results in more pronounced circadian misalignment compared with westward travel, particularly during long-haul travel involving multiple timezones, because the phase advance required for re-entrainment is more difficult to achieve than the phase delay required when traveling west [74]. Beyond direction and duration of travel, the context or purpose of travel may also influence the severity and persistence of jet-lag symptoms [75]. Nonetheless, substantial travel in either direction can meaningfully disrupt sleep; for example, NCAA Division 1 female volleyball players traveling westward across nine timezones exhibited significant disturbances in sleep patterns, which may impair athletic performance [76]. In another study, athletes traveling eastward took approximately 1–2 additional days to resolve jet-lag symptoms compared with westward travel [76, 77]. The effects of circadian phase disruptions and jet lag on performance have been reported in many sports including baseball, basketball, football, and swimming [22, 78–83]. All studies show negative consequences of trans-timezone travel but differ as to whether eastward or westward travel is worse, often depending on the time of day of travel and the time of competition. The disadvantage of westward travel is that the resulting jet lag can put athletes in non-optimal times for specific components of performance, especially for games timed at the traveling team’s bedtime.

In a population-level analysis of elite international competitors, eastward travel across multiple timezones was associated with greater performance decrements, reflected by shifts in medal distributions including a higher likelihood of athletes expected to win gold placing lower on the podium (the “gold demotion” effect); however, these associations were observed in elite competitive contexts and should not be assumed to generalize to non-elite athletes or to predict individual outcomes [81]. Additionally, rapid travel across timezones, regardless of direction, can lead to jet lag, sleep deprivation, and other physiological stressors that negatively impact athletic performance [1, 84].

The normal phase adjustments required for daily entrainment of circadian body rhythms to the 24-h day length are in the order of minutes, whereas phase adjustments following east–west travel span 1 h or more. Given that humans can shift their circadian rhythms by approximately 1 h per day depending on the magnitude and timing of light exposure, crossing even a few timezones requires multiple days to fully re-entrain [23, 85, 86].

To minimize the adverse effects of westward travel, athletes can consider the following strategies: (1) Gradual schedule adjustment: before traveling, gradually shift sleep to align more closely with the destination’s timezone. ​(2) Light exposure management: light use should be timed according to the individual’s current circadian phase and the number of timezones crossed. For shorter westward trips, evening light at the destination helps delay the circadian phase; for travel across many timezones, evening light may paradoxically advance the circadian phase, hindering adaptation [20].​ (3) Melatonin supplementation: melatonin can assist with circadian re-entrainment, but timing of administration relative to the current circadian phase is critical, as it can either promote or oppose the desired phase shift [20]. (4) Hydration and nutrition: maintaining proper hydration and adjusting meal times to the destination’s schedule can support overall well-being and adaptation [20].​

### Athlete Chronotypes

Athlete chronotype and diurnal preference could also play a role in optimizing performance and recovery during travel. While often used interchangeably, chronotype is often used as a behavioral proxy to reflect individual differences in circadian biology, while diurnal preference refers to one’s preference towards the morning or evening [87, 88]. Chronotypes are typically classified as morning types (early chronotypes), evening types (late chronotypes), or intermediate types. These groups reflect individual variations in many physiological processes including sleep–wake patterns, peak performance times, and hormonal rhythms such as melatonin and cortisol [89, 90]. The gold standard method for assessing objective circadian phase is dim light melatonin onset, but some non-invasive methods to identify chronotype include questionnaires, such as the Munich Chronotype Questionnaire and the Morningness-Eveningness questionnaire for diurnal preference [91–93].

Understanding an athlete’s chronotype could inform personalized strategies for maintaining timezone alignment or phase shifting the circadian system to optimize performance. However, these strategies would need to be sport-specific, as the logistical aspects of team sports can make individualized chronotype-based scheduling challenging. In team based sports where athletes must train and travel together, considering the composition of chronotypes within the team could provide some insight into group level strategies [94]. Additionally, there are inconsistencies in the literature, with some recent studies suggesting chronotype does not play a role in the adjustment to long-haul travel-induced jet lag, and others suggesting that it does [20, 29]. One of the major limitations in the literature on jet lag in athletes is the small sample size, lack of variability in chronotype groups and differing methods (if any) to assess chronotype [28, 95]. The general hypothesis for the influence of chronotype on jet lag is based on research on social jetlag and night shift workers [20, 96]. This suggests that early chronotypes may perform better in early competitions and adapt more quickly to eastward travel, which involves phase advancing the circadian system [90]. In contrast, late chronotypes may excel in evening/night competitions and adapt more readily to westward travel, which requires phase delaying the circadian system [97]. Tailoring travel schedules, training sessions, and competitions to align with an athlete’s chronotype could enhance performance and reduce the risk of travel-related disruptions. Additionally, interventions such as strategic light exposure, modified sleep patterns, timed use of exogenous melatonin and chronotype-specific meal timing can further support circadian alignment during timezone transitions [98].

## Maximizing Physiology for Performance

Maximizing physiology for athletic performance is important to consider when traveling. Specific strategies include optimizing sleep, light exposure, fueling, and supplementation (Table 2).

**Table 2:** Interventions to maximize physiology for performance in athletes

Intervention	Timing/phase	Practical implementation tips
Bright light exposure	After arrival: biological morning for eastward, late biological afternoon/night for westward travel	Spend 30–180 min in natural light. Use light boxes if needed. Avoid sunglasses during planned exposure. Use publicly available tools (e.g., www.timeshifter.com) before, during, and after travel for personalized, evidence-based light exposure timing recommendations
Light avoidance	After arrival: during biological afternoon/night for eastward, during biological morning for westward	Use eye masks, dim lighting, or blue-blocking glasses. Stay indoors during inappropriate light windows
Melatonin supplement	30–60 min before local bedtime (first 2–5 nights)	Dose: 2–5 mg immediate release. Take in a dim setting. Avoid daytime use. Start low for sensitive individuals
Strategic napping	During travel and early after arrival (early afternoon)	Keep naps 20–30 min. Avoid evening naps. Use eye masks/earplugs. Optional longer nap (~ 90 min) if extremely fatigued
Caffeine use	Morning to early afternoon at destination	Use ~ 100–200 mg to boost alertness. For competition performance, consider typical ergogenic dosing (~ 3–6 mg/kg ~ 60 min pre-event) when appropriate and trial in training; avoid or reduce caffeine for late-day/evening events to protect sleep. Avoid caffeine within ~ 8–10 h of target bedtime. Consider gum or tea as alternatives
Sleep hygiene	Night before, during travel, and every night after arrival	Cool, dark, quiet room. Use earplugs, eye masks. Limit screens and avoid large meals within ~ 2–3 h of bedtime (especially high-fat/spicy foods). Maintain a familiar bedtime routine
Gradual time shift	3–7 days pre-travel	Shift sleep/wake times by 30–60 min/day toward destination. Match mealtimes. Ensure adequate sleep throughout
Sleep banking	3–5 days pre-travel	Aim for 8–9 + h/night. Add naps if needed. Helps buffer sleep loss during travel
Exercise timing	After arrival: biological morning for eastward, late biological afternoon/night for westward travel	Light/moderate activity (e.g., walk, jog). Avoid intense workouts within 2–3 h of bedtime
Meal timing and nutrition	During and after travel	Eat meals aligned with local time as soon as possible. Favor light, digestible foods. Stay hydrated. Avoid sugar/caffeine near bedtime

### Considerations on Sleep Consistency, Sleep Duration, and Intermittent Napping

Athletic performance is regulated by multiple interdependent physiological systems, many of which are modulated by sleep. In elite sport, travel and competition schedules frequently disrupt circadian rhythms, impair sleep continuity, and attenuate recovery processes. These disruptions are associated with reduction in cognitive performance, metabolic efficiency, immune function, and neuromuscular output [5, 99].

#### Sleep Consistency

Sleep consistency, defined broadly as the day-to-day regularity of sleep onset and wake times, is strongly associated with improved sleep efficiency, greater alignment of endogenous circadian rhythms, and enhanced autonomic balance [100]. Air travel across timezones may disrupt sleep consistency by altering habitual sleep schedules and delaying circadian re-entrainment, resulting in irregular sleep timing during the days following travel [101]. Greater variability in sleep timing has been linked to decreased cognitive performance, elevated resting heart rate, and reduced heart rate variability – markers of compromised physiological readiness [102, 103].

Winter et al. introduced the concept of “circadian advantage”, defined as the relative difference between competing teams in their degree of circadian misalignment at the time of competition, based on the number of timezones crossed and days since travel [8]. Using a 10-year retrospective analysis of Major League Baseball games, teams with greater circadian advantage, that is, closer circadian alignment relative to their opponent, were more likely to win, with effects increasing as the magnitude of circadian advantage increased. This framework highlights how circadian misalignment following travel can influence competitive outcomes when teams differ in their degree of resynchronization. Although sleep consistency was not directly measured, circadian advantage likely reflects differences in sleep timing regularity and circadian alignment following travel.

Emerging evidence also supports associations between sleep consistency and individual-level athletic performance. In elite golfers, both between-athlete differences and within-athlete improvements in sleep consistency were associated with better objective performance outcomes, including lower scores, highlighting the relevance of sleep-timing regularity in precision-based sports [104].

#### Sleep Duration

Athletes often experience reduced sleep duration when traveling, particularly across multiple timezones. This reduction is primarily due to jet lag and circadian rhythm disruptions. While specific studies quantifying the exact amount of sleep loss are limited, research indicates that even minor timezone changes can significantly impact sleep quality and duration.​ Sleep duration is foundational for neuroendocrine regulation, synaptic plasticity, memory consolidation, and musculoskeletal repair [105, 106]. A study by Waterhouse et al. highlighted that athletes traveling across timezones often suffer from sleep disturbances, leading to decreased performance [3]. The study emphasized the importance of considering travel-induced sleep disruptions when planning training and competition schedules. Experimental evidence further supports the critical role of sleep duration in athletic performance. For example, a recent systematic review and meta-analysis demonstrated that acute sleep deprivation impairs performance across multiple domains, including high-intensity exercise, skill execution, speed, aerobic capacity, endurance, and explosive performance [107].

Beyond the travel context, habitual sleep sufficiency plays a critical role in athletic performance.

Both acute and chronic sleep restriction have been shown to degrade reaction time, decision making, glucose metabolism, and exercise capacity [27, 108]. Conversely, athletes who consistently meet or exceed baseline sleep requirements (i.e., 7–9 h per night) demonstrate improvements in objective performance measures and subjective recovery, as shown in sleep extension studies conducted outside of travel settings [105].

#### Intermittent Napping

Intermittent napping serves as an auxiliary strategy to compensate for sleep debt and attenuate performance decrements due to partial sleep loss. Short and long naps (10–90 min) have been shown to restore alertness, enhance sprint performance, and improve cognitive throughput following sleep restriction [109–111]. Collectively, these studies suggest that strategic napping can be an effective countermeasure against performance decrements due to sleep loss in athletes. The timing and duration of naps are also critical, with optimal performance benefits observed when naps occur in the early-to-mid afternoon (e.g., 1–4 p.m.), lasting 20–90 min, to enhance physical and cognitive functions [111–114]. Interestingly, evidence suggests that both short (20–30 min) and longer (> 35–90 min) naps may enhance or restore physical and cognitive performance, although longer naps may confer greater benefits in some contexts [114]. However, sufficient time after waking should be allowed before engaging in training or competition to reduce sleep inertia.

### Light Exposure

Light is the primary and most powerful *zeitgeber*, or time-giver, referring to an external environmental cue that synchronizes internal circadian rhythms to the 24-h day [115, 116]. Zeitgebers provide temporal information to the circadian system, allowing endogenous rhythms to align with predictable environmental rhythms like the light–dark cycle. Predictably timed photic input via the retina resets the central circadian clock in the suprachiasmatic nucleus (SCN) which prepares and facilitates appropriately timed physiological, cognitive, and behavioral functioning [117, 118]. This light-sensitive circadian system evolved under consistent and predictable light–dark conditions, and therefore travel across timezones that is accompanied by rapid changes to light–dark conditions can lead to significant circadian disruption and compromise circadian-regulated physiological functioning [119].

Appropriate light exposure and light avoidance can accelerate re-entrainment of circadian rhythms to the destination timezone and shorten the symptomatic “jet lag” period [120, 121]. Phase-response curves (PRCs) have been developed to characterize the appropriate timing, illuminance, spectrum, and duration of light exposure to produce the desired phase shifts [122–125]. Although PRCs were developed under highly controlled laboratory conditions and their practical implementation in real-world travel scenarios is often limited by variable ambient light exposure and reduced environmental control, they remain the strongest framework available for guiding light-based counter-measures against jet lag in athletes.

Consistent with the human phase response curve to light, eastward travel (requiring a phase advance) is generally supported by bright light exposure during the late biological night to early biological morning, typically in the hours immediately following the core body temperature minimum (CBTmin), whereas westward travel (requiring a phase delay) is supported by bright light exposure during the early biological night, in the hours preceding CBTmin [86, 120]. Importantly, biological time should not be conflated with local destination clock time, as doing so may result in light exposure producing the opposite of the intended effect. For example, following eastward travel from Boston to London (and thus requiring a phase advance), exposure to bright light in the early London morning may delay the athlete’s circadian clock, because this clock time can coincide with the athlete’s early biological night.

Given the complexity of estimating individual circadian phase and dynamically updating light exposure recommendations across days of travel, athletes and support staff may benefit from structured, evidence-based decision tools. Several publicly available tools provide step-by-step guidance on light exposure based on travel schedules and prior sleep–wake behavior (e.g., TimeShifter; www.timeshifter.com).

### Exercise Timing, Duration, and Intensity

In addition to light, there is evidence that exercise is a zeitgeber capable of influencing circadian rhythmicity, making it a potential countermeasure for athletes adapting to east–west travel [126–128]. Consistent with bright light PRCs, an exercise PRC suggests exercise performed during the early biological morning can produce a phase advance, whereas exercise performed during the early biological night can produce a phase delay [126]. Therefore, strategically timed exercise may complement light exposure interventions following eastward and westward travel.

Importantly, the duration and intensity of exercise are also associated with disruptions to subsequent sleep. Recent large-scale analyses demonstrate evening exercise is dose dependently associated with disruptions to sleep and autonomic activity such that the more strenuous and later the evening exercise is, the worse the associated disruptions are [129]. For travelling athletes, these findings suggest exercise timing, particularly for training sessions that are modifiable, should be individualized and context-specific. Appropriately timed exercise may support circadian re-entrainment without compromising sleep, while strenuous exercise sessions should be scheduled earlier in the biological day – particularly after eastward travel.

### Fueling

Proper nutrition is essential for maintaining athletic performance, health, and readiness throughout all phases of travel. Although individual macronutrient needs vary based on sports type, training status, and season, travel presents common challenges such as reduced appetite, GI distress, unfamiliar foods, and disrupted meal timing, which can compromise overall energy intake and composition [130].

Maintaining sufficient energy availability while traveling is important, as even short periods of underfueling may impair muscle glycogen stores, recovery, and immune function [131–133]. For example, just 3 days of typical training under low energy availability resulted in a ~ 30% reduction in muscle glycogen compared to normal energy availability [132]. Acute energy availability deficits are also associated with impairments in myofibrillar and sarcoplasmic muscle protein synthesis, which may negatively affect skeletal muscle adaptation, although muscle strength may be preserved [134, 135]. Over time, repeated episodes of inadequate fueling, such as those encountered across a competitive season with frequent travel, may increase the risk of chronic energy deficiency with downstream effects on performance, endocrine and bone health, and immune function [136]. Prophylactically, athletes should begin travel in a well-fueled state, and carry easy-to-digest and energy-dense snacks to help mitigate the risk of energy deficits.

GI distress such as bloating, nausea, constipation, and diarrhea is commonly reported during athletic travel. Mechanistically, circadian misalignment can alter GI motility and digestive enzyme secretion, contributing to symptoms [137, 138]. To support GI homeostasis, athletes are encouraged to choose familiar and easily digestible foods before and during flights. Additional strategies include avoiding large meals in favor of smaller, more frequent snacks, maintaining hydration, and limiting excessive fiber intake during travel [139].

Meal timing and composition are other potential tools for supporting circadian alignment and performance during travel, as reviewed in detail elsewhere [139]. While the central circadian pacemaker resides in the SCN of the anterior hypothalamus, peripheral clocks in metabolically active tissues, such as the liver, are sensitive to meal timing and composition [140]. Evidence from animal studies suggests that timed feeding can help reset peripheral clocks following travel, and emerging human studies support similar benefits [141, 142]. Contemporary sports nutrition evidence supports maintaining adequate energy balance during travel with appropriately timed carbohydrate and protein consumption to support both performance and sleep [139]. Accordingly, fueling strategies during travel should prioritize consistency, digestibility, and energy availability.

Regarding dietary composition, data in the context of travel are limited, but nutritional strategies to support sleep in athletes may offer indirect benefits [143]. Several dietary components may influence neurotransmitter synthesis and sleep architecture. For example, tryptophan, a precursor to serotonin and melatonin, crosses the blood–brain barrier more readily in the presence of elevated insulin following carbohydrate intake [144]. Evening meals containing tryptophan-rich proteins alongside carbohydrates may therefore support sleep onset. Supporting this, high-glycemic index meals consumed 4 h before bedtime have been demonstrated to reduce sleep latency [145]. However, high carbohydrate intake may also be associated with less slow-wave sleep and greater REM sleep, potentially from increased glucose demands during elevated neural activity [146]. Conversely, low-carbohydrate meals before bed have been associated with increased restorative sleep compared to control [147].

To optimize readiness in the traveling athlete, fueling strategies that maintain energy availability, support GI tolerance, and align with circadian rhythms should be prioritized. Practical approaches to mitigate travel-related stress include packing familiar snacks, adjusting meal timing, and choosing compositionally appropriate meals.

### Supplements

Dietary supplement use is widespread, with more than half of US adults reporting regular consumption, and use rates as high as 96% in athletic populations [148, 149]. In the context of travel, a systematic review and meta-analysis identified 12 functional foods, beverages, and supplements that may help to mitigate air travel symptoms [150]. In the present article, we focused on those most relevant to athletes, including melatonin, caffeine, macronutrient composition, fluid and hydration strategies, and dietary fiber. Among these, melatonin and caffeine have received the greatest attention for their potential roles in mitigating the negative effects of jet lag and performance impairments. The following section examines the evidence supporting these agents as targeted interventions to support athletes during travel.

#### Caffeine

Caffeine is one of the most widely consumed supplements that elevates alertness and cognitive function by antagonizing adenosine receptors and stimulating the sympathetic nervous system [151]. In the context of athlete travel, strategically timed caffeine may attenuate jet-lag-induced performance decrements, and maintain vigilance through extended wakefulness and circadian rhythm retraining following time change [152, 153]. In a recent randomized controlled trial in jet-lagged ice hockey players, moderate-dose caffeine (3 mg/kg body weight) improved explosive power via vertical jump compared to placebo after an eastward travel simulation [154]. Importantly, moderate caffeine consumption does not appear to contribute to dehydration, providing reassurance for concerns relating to fluid balance during travel. Consensus guidelines from the International Society of Sports Nutrition recommend doses of 3–6 mg/kg, administered ~ 60 min before exercise, as effective, safe, and well tolerated for enhancing athletic performance [48, 155, 156]. However, both timing and dose are important as caffeine consumed later in the day or during the circadian night, particularly at higher doses, can delay circadian re-entrainment and impair subsequent sleep [157]. A recent randomized controlled trial demonstrated that 400 mg of caffeine taken within 12 h of bedtime delayed sleep onset, disrupted sleep architecture, increased sleep fragmentation, and reduced subjective sleep quality [158]. Although not traditionally classified as a chronobiotic, emerging evidence suggests that caffeine can modestly shift circadian rhythms, particularly when consumed in the evening by delaying melatonin onset [159]. Collectively, the evidence suggests caffeine may serve as a useful tool for maintaining alertness and performance during travel when carefully timed earlier in the day, while strategies to limit or taper caffeine intake and support subsequent sleep, such as dietary interventions with sleep-promoting properties (e.g., tart cherry products), may help preserve sleep and circadian alignment [160, 161].

#### Magnesium

Magnesium is the fourth most abundant mineral in the body and serves as a cofactor in over 300 enzymatic reactions, including those involved in energy metabolism, neuromuscular function, bone integrity, immune defense, pain modulation, and regulation of stress [162, 163]. While athletes may have higher intakes compared to the general population, they may still be at risk for magnesium deficiency due to increased losses through sweat and urine [164].

In the context of travel, magnesium has drawn interest for its potential to support sleep and circadian regulation. Mechanistically, magnesium modulates activity of gamma-aminobutyric acid (GABA), a key inhibitory neurotransmitter, and influences melatonin synthesis and receptor binding [165, 166]. These actions may explain observational findings linking higher magnesium status to better sleep quality and duration [167]. Other studies have yielded more mixed results, likely due to heterogeneity in study populations (e.g., magnesium sufficiency at baseline), measured outcomes (e.g., cognitive function, sleep), and supplementation protocols (e.g., type, dose, timing) [167].

Recent evidence suggests magnesium supplementation is beneficial for treatment of mild anxiety and insomnia, with greater effects observed among those with low baseline magnesium status [168]. Among the various formulations, magnesium L-threonate has garnered particular attention due to its unique ability to cross the blood–brain barrier and effectively elevate blood magnesium levels [169]. Preliminary human trials have demonstrated potential cognitive and sleep-related benefits with this form, although studies in athletes remain limited. Magnesium L-threonate may represent a promising strategy to support cognitive function, sleep quality, and circadian alignment in the traveling athlete, but further research is needed [170, 171].

#### Melatonin

Melatonin is the primary hormone regulating the sleep–wake cycle and functions as a central chronobiotic, aligning the internal circadian clock with the external light–dark cycle [172]. Under normal conditions, melatonin secretion follows a diurnal rhythm, rising in the evening to facilitate sleep onset, reaching acrophase (peak) during the night, and declining in the morning to promote wakefulness. Transmeridian travel disrupts this rhythm, contributing to jet-lag symptoms including sleep disruption, daytime fatigue, and reduced alertness [173]. Accordingly, exogenous melatonin supplementation has been widely studied as a countermeasure to facilitate circadian re-entrainment following timezone shifts.

A meta-analysis in individuals with primary sleep disorders demonstrated that melatonin supplementation reduces sleep-onset latency and increases total sleep time and sleep duration across both adult and pediatric populations [174]. Additional meta-analyses support melatonin’s efficacy in reducing sleep latency among adults with comorbid insomnia and children and adolescents with mental health disorders, though no benefits were observed for total sleep time, sleep efficiency, or number of awakenings [175, 176]. In healthy individuals, particularly outside the context of travel or circadian disruption, evidence remains limited. While melatonin has been shown to increase subjective sleepiness in healthy young adults, low doses (0.3 or 1.0 mg taken 2–4 h prior to bedtime) do not appear to alter sleep architecture when measured by polysomnography [177, 178]. In contrast, a randomized controlled trial in an international flight crew demonstrated that 5 mg of melatonin improved self-reported jet-lag symptoms and sleep quality [179]. Among athletes, high-dose melatonin supplementation (5–100 mg) has been examined primarily in the context of modulating biomarkers of inflammation and oxidative stress, rather than for circadian phase shifting or sleep outcomes [180]. Importantly, these doses exceed those typically required to induce circadian phase shifts, which are generally achieved at much lower doses administered at appropriately timed intervals relative to the desired sleep schedule [181]. Furthermore, high doses may result in melatonin remaining in circulation for a prolonged duration, which could increase the likelihood that it will act upon the opposite portion of the phase-response curve and thereby attenuate the desired phase-shifting effect [182]. As such, extrapolation of findings from high-dose melatonin studies to the management of jet lag or circadian re-entrainment should be approached with caution, as excessive dosing may blunt or misalign circadian signaling rather than facilitate adaptation [183].

Despite the variability in findings, melatonin’s relatively low side-effect profile and mechanistic role in circadian regulation support its consideration as a potentially effective and well-tolerated intervention to mitigate circadian disruption associated with sports-related travel. Further research is needed to determine optimal dosing strategies and to evaluate efficacy in athletic cohorts.

## Environmental Considerations

Athletes frequently travel domestically and internationally for training camps and competitive events, exposing them to environmental stressors such as high altitude, extreme heat, and humidity – conditions to which they are often not adequately acclimated or acclimatized. These stressors have been reported anecdotally and scientifically to impose significant physiological and cognitive demands, potentially impairing athletic performance. Additionally, travel-related fatigue and stress further compound these challenges, hindering optimal performance upon arrival. This section critically examines the physiological impacts of these special environmental circumstances on traveling athletes and discusses evidence-based strategies aimed at mitigating their detrimental effects.

### Altitude

Training or competing at high altitude (≥ 1500–2000 m) exposes athletes to reduced barometric pressure and a lower partial pressure of inspired oxygen (PiO₂), resulting in systemic hypoxia. This reduced oxygen availability triggers increased sympathetic activity, elevated ventilation, and higher heart rate to maintain oxygen delivery [184]. Hypoxia also stimulates erythropoietin (EPO) production from renal peritubular fibroblasts, promoting erythropoiesis to enhance oxygen-carrying capacity [185, 186]. Although EPO levels rise within hours, significant increases in hemoglobin mass require several weeks of sustained exposure [187]. During early acclimatization, athletes may experience fatigue, reduced aerobic capacity, and impaired performance until these adaptations take effect.

Air travel introduces additional altitude-related stress. Aircraft cabins are typically pressurized to the equivalent of ~ 2,440 m with low humidity (< 20%), leading to mild hypoxia and increased respiratory water loss [188]. These conditions, combined with limited fluid intake, often result in subclinical hypohydration that can impair performance upon arrival.

Hydration becomes critical at altitude, where low humidity and elevated ventilatory drive increase respiratory fluid loss. Additionally, altitude-induced diuresis exacerbates dehydration risks, leading to reductions in plasma volume that compromise cardiovascular stability and thermoregulation [189]. When combined with heat exposure, sweat loss and electrolyte depletion further elevate physiological strain [45]. Proactive hydration is essential to maintain plasma volume and performance.

Altitude also disrupts sleep. Periodic breathing is characterized by cycles of hyperventilation and apnea, which results in fragmented sleep, breathlessness, and poor recovery [190, 191]. These disruptions can impair cognition, mood, and athletic performance, especially when compounded by chronic hypoxic exposure.

Nutritionally, altitude suppresses appetite despite an elevated metabolic rate, increasing the risk of negative energy balance. Inadequate intake may lead to lean mass loss, reduced glycogen stores, and impaired recovery [192].

Endurance performance is most affected, with decreased VO₂_max_, elevated perceived exertion, and reduced aerobic capacity [184]. While short-duration anaerobic efforts may benefit from reduced air resistance, any advantage is often offset by generalized fatigue and poor recovery [193].

Jet lag further compounds altitude stress. Circadian misalignment and sleep disruption are intensified under hypoxia, impairing reaction time, cognitive function, and immune resilience [1]. Without adequate acclimatization, the combined burden of hypoxia, dehydration, poor sleep, and jet lag can substantially impair athlete health and performance [20].

#### Mitigation Strategies for Altitude

Acclimatization represents an essential strategy for mitigating the adverse physiological effects associated with altitude exposure in traveling athletes. Current guidelines suggest that athletes traveling to moderate altitude (approximately ≤ 2500 m) should arrive at least 2 weeks prior to competition to facilitate adequate physiological adjustments, such as enhanced erythropoiesis, improved oxygen-carrying capacity, and progressive normalization of elevated resting heart rate and ventilation rates [184, 189]. For destinations above 2500 m, gradual ascent at a controlled rate of approximately 600–1200 m per day is recommended to minimize the risk of acute altitude illnesses, including acute mountain sickness (AMS), and to allow sufficient adaptation time to reduce the partial pressure of inspired oxygen [189].

Normobaric hypoxia offers a practical preconditioning strategy to enhance altitude tolerance before travel. Using altitude tents or hypoxic chambers to reduce inspired oxygen at sea-level pressure, athletes can simulate moderate altitude and induce adaptive responses. These include increased erythropoietin production, red blood cell mass, and oxygen-carrying capacity [194]. Additional benefits include improved mitochondrial efficiency, muscle buffering, and upregulation of hypoxia-inducible factor-1α (HIF-1α), which mediates cellular adaptation to hypoxia [195, 196]. Intermittent hypoxia training (IHT) may further enhance ventilatory and metabolic adaptation with less logistical demand [197]. Though individual responses vary, normobaric hypoxia provides a feasible, evidence-based method to precondition athletes for altitude exposure.

Altitude blunts thirst perception and increases fluid loss via elevated ventilation and altitude-induced diuresis, making proactive hydration essential. Athletes should begin altitude exposure euhydrated and consume fluids regularly (e.g., ~ 6 mL·kg⁻^1^ every 2–3 h), with sodium-containing beverages to support plasma volume, particularly when altitude exposure coincides with travel or heat stress [45, 184, 189].

### Heat and Humidity During Travel

Athletes traveling to international competitions in hot, humid climates face compounded physiological challenges from both environmental heat stress and travel-related disruptions such as jet lag and circadian misalignment. Heat and humidity significantly impair thermoregulation, which is already compromised by the fatigue, sleep disruption, and altered routines that accompany long-haul travel.

In high ambient temperatures, thermoregulation requires increased skin blood flow and sweating to dissipate heat. This induces cardiovascular strain by reducing central blood volume and redirecting blood flow away from working muscles; effects that are intensified by dehydration and the cardiovascular fatigue often associated with long flights [198–200]. The resulting cardiovascular drift elevates heart rate for a given workload, decreasing efficiency and increasing perceived exertion.

Exercise in the heat accelerates core body temperature rise (hyperthermia), which impairs performance and increases the risk of heat illness. These effects are magnified in humid environments where evaporative cooling is less effective [201, 202]. During travel to such climates, the body may not yet be acclimated, increasing susceptibility to heat-related fatigue and illness.

Heat stress also alters substrate metabolism, increasing muscle glycogenolysis and whole-body carbohydrate oxidation, a shift amplified in females and accelerated muscle tissue temperature rises [203–205]. When combined with dehydration, which is common during and after air travel, these shifts lead to faster glycogen depletion and earlier onset of fatigue.

Dehydration is exacerbated in hot climates due to increased sweat loss, and even mild deficits (~ 2% body mass) impair aerobic capacity, cognitive function, and thermoregulatory efficiency [42, 202]. If not addressed upon arrival, the combined effects of heat, humidity, and travel fatigue heighten the risk of exertional heat illness and undermine performance readiness.

Given the compounded effects of heat stress, humidity, and travel-related hypohydration, athletes should implement a simple hydration strategy upon arrival in hot environments. This includes prioritizing early rehydration with fluids containing sodium, consuming fluids at regular intervals rather than ad libitum, and monitoring urine color and body mass changes during the first 24–48 h post-travel [42, 202]. Initiating training or competition in a euhydrated state is critical, as even mild hypohydration can accelerate core temperature rise, increase cardiovascular strain, and impair thermoregulatory efficiency in hot and humid conditions.

#### Effects of Heat and Humidity on Sleep and Recovery

Heat and humidity disrupt sleep architecture by impairing the normal decline in core body temperature required for initiating and maintaining deep, restorative sleep [206]. Without adequate night-time cooling or climate control, elevated ambient temperature and humidity reduce evaporative heat loss, lowering thermal comfort and increasing sleep fragmentation – particularly reductions in slow-wave sleep [207]. These disturbances compromise recovery, cognitive function, and overall performance, especially when layered onto travel-related circadian disruption.

Unacclimated athletes exposed to hot, humid environments experience reduced endurance capacity due to impaired thermoregulation, earlier onset of fatigue, and slower recovery between efforts. While sprint and power-based performance is less directly impacted, cumulative heat strain, dehydration, and neuromuscular fatigue can still impair output [208]. Athletes with high aerobic fitness or prior heat acclimatization demonstrate more efficient thermoregulation, including earlier sweat onset, better fluid balance, and maintained plasma volume, enhancing resilience to heat stress [200, 202].

#### Combined Effects of Heat, Humidity, and Jet Lag on Athlete Hydration and Performance

The convergence of heat, humidity, and jet lag creates a high-risk environment for dehydration and performance impairment in traveling athletes. Long-haul flights commonly induce subclinical dehydration due to low cabin humidity, prolonged immobility, and limited fluid intake [45]. Upon arrival in a hot climate, athletes often begin training or competition in a hypohydrated state, which accelerates core temperature rise and increases cardiovascular strain during exercise due to reduced plasma volume and impaired thermoregulation [42, 202]. Jet lag and circadian misalignment can blunt thirst perception and suppress voluntary fluid intake, particularly within the first 24–48 h post-travel [45]. This further delays rehydration and increases the likelihood of entering physical exertion with inadequate fluid reserves, exacerbating symptoms such as fatigue, reduced mental clarity, and impaired exercise tolerance. In some competition settings, heat stress may coincide with moderate altitude exposure. Heat elevates sweat loss and cardiovascular load, while altitude reduces arterial oxygen saturation and increases ventilatory demand. When dehydration is superimposed, the compounded reduction in plasma volume diminishes both thermoregulatory efficiency and oxygen delivery, significantly impairing endurance performance and increasing the risk of exertional heat illness [193].

### Mitigation Strategies for Heat Exposure

Mitigating heat strain requires a combination of heat acclimatization and structured fluid management, as emphasized by Racinais et al.[202]. Heat acclimatization involves 7–14 days of daily exercise in hot conditions (≥ 60 min), inducing sustained thermal strain (elevated core temperature and sweating) to drive adaptations such as plasma volume expansion, earlier sweat onset, increased sweat rate, and improved cardiovascular stability [200]. These adaptations enhance heat dissipation, lower thermal and cardiovascular strain, and improve exercise capacity in the heat. When environmental access is limited, heat stress can be simulated using impermeable clothing or post-exercise sauna bathing, which promote similar thermoregulatory responses [209]. The majority of adaptations develop within the first 5–7 days, with further gains over 10–14 days. Benefits can persist for 2–4 weeks but gradually wane without continued heat exposure, highlighting the need for maintenance sessions or re-acclimatization before competition [210].

#### Hydration Strategies for Hot and Humid Environments

Attempting euhydration is vital for sustaining thermoregulation and performance in hot, humid conditions, where sweat losses increase markedly [42]. During acclimatization and training preparation, ~ 6 mL of fluid per kg body mass every 2–3 h is recommended [211]. Given wide individual variation in sweat rate (~ 1.0–2.5 L/h), athletes should personalize intake by tracking body mass changes pre- and post-exercise. Following sessions > 60 min, rehydration should target 120–150% of body mass lost and include sodium (20–50 mmol/L) to support fluid retention and restore electrolyte balance [212]. Adding carbohydrates (6–8%) to fluids may further enhance fluid absorption and assist with glycogen replenishment during prolonged efforts.

#### Cooling Strategies to Mitigate Heat Strain and Support Recovery

Effective cooling interventions are essential for reducing heat strain, sustaining performance, and enhancing recovery in hot conditions. These strategies lower core and/or skin temperature, help maintain central blood volume, and improve thermal comfort, collectively delaying fatigue and reducing physiological stress [213, 214]. Phase-specific approaches are outlined in Table 3. Pre-cooling increases heat storage capacity, mid-exercise cooling mitigates thermal load and perceptual strain, and post-exercise cooling accelerates recovery and inflammation resolution. Additionally, heat exposure can disrupt the core temperature decline needed for sleep, impairing sleep quality and subsequent recovery.

**Table 3:** Summary of phase cooling strategies for athletes

Phase	Strategy	Protocol/modality	Key effects	Key reference
Pre-exercise	Cold water immersion	14–18 °C immersion for 10–20 min	Lowers core temp; enhances endurance in the heat	Bongers et al., 2015 [213]
	Ice vests/phase-change garments	Wearing cooling garment for 10–20 min	Reduces skin temp; lowers perceived strain; ↑ time-to-exhaustion	Duffield et al., 2013 [221]
	Topical cooling agents	Menthol spray/gel applied to skin	Improves thermal comfort; minimal core temp change	Stevens et al., 2016 [222]
During exercise	Cold fluid ingestion	Drink 4–10 °C fluids ad libitum	Modest core temp ↓; better hydration and comfort	Lee et al., 2008 [223]
	Ice slurry ingestions	Ingest semi-frozen slurry periodically	Greater cooling than cold fluids; prolongs performance	Siegel et al., 2010 [224]
	Menthol mouth rinse	5- to 10-s rinse with menthol solution every 15–20 min	Strong perceptual cooling; ↑ endurance under heat stress	Gavel et al., 2024 [225] Hawke et al., 2025 [226]
	Environmental and local cooling	Shade, water dousing, ice towels during breaks	Lowers skin temp; ↓ perceptual load	Tyler et al., 2015 [214]
Post-exercise	Cold water immersion	10–15 °C immersion for 10–15 min	Rapidly ↓ core temp; aids neuromuscular recovery	Poppendieck et al., 2013 [227]; Cain et al., 2025 [217]
	Cold showers/contract water	Alternate or cold shower for 5–10 min	Accessible cooling; ↓ skin and muscle temp	
Sleep	Optimize sleep environment	Fans/air conditioning; light, moisture-wicking bedding	Enhances sleep initiation and quality in heat	Okamoto-Mizuno & Mizuno, 2012 [207]; Chevance et al., 2024 [218]; Li et al., 2025 [219]
	Pre-sleep behavior	Avoid intense exercise ≤ 2 h before bed	Prevents residual core temp elevation	Leota et al., 2025 [129]
	Circadian support	Morning bright light; limit blue light before bed	Realigns circadian rhythm; improves recovery	Burgess et al., 2003 [215]

In addition to reducing thermal strain during exercise, cooling strategies may support post-exercise recovery by facilitating the decline in core body temperature required for sleep initiation. Contemporary sleep research demonstrates that elevated night-time core and skin temperatures impair sleep onset, reduce slow-wave sleep, and increase sleep fragmentation – effects that are exacerbated following heat exposure and late-day exercise. Accordingly, post-exercise and pre-sleep cooling strategies may serve a dual role by attenuating physiological heat strain while supporting sleep quality and recovery in hot environments [129, 212, 215–219].

## Conclusion

Athletic travel presents a multifactorial stressor with significant implications for performance, health, and recovery. Evidence-based strategies targeting hydration, nutrition, circadian alignment, sleep, and gut health can potentially mitigate these effects. Individualized interventions that consider athlete chronotype and sport-specific demands may further enhance outcomes. Additional considerations include optimizing travel logistics and leveraging tools such as light exposure, supplements, and schedule adjustments. As global sport continues to evolve and international competition becomes increasingly common, travel must be treated as a core component of performance planning.

## Declarations

### Availability of Data and Material

Not applicable. This review did not generate any data.

## References

[1] Leatherwood WE, Dragoo JL. Effect of airline travel on performance: a review of the literature. Br J Sports Med. 2013;47(9):561–7. 10.1136/bjsports-2012-091449. 23143931 10.1136/bjsports-2012-091449

[2] Lee A, Galvez JC. Jet lag in athletes. Sports Health. 2012;4(3):211–6. 10.1177/1941738112442340. 23016089 10.1177/1941738112442340 PMC3435929

[3] Waterhouse J, Reilly T, Atkinson G, Edwards B. Jet lag: trends and coping strategies. Lancet. 2007;369(9567):1117–29. 10.1016/S0140-6736(07)60529-7. 17398311 10.1016/S0140-6736(07)60529-7

[4] Botonis PG, Toubekis AG, Hill DW, Mündel T. Impact of long-haul airline travel on athletic performance and recovery: A critical review of the literature. Exp Physiol. 2025. 10.1113/EP091831. 40121547 10.1113/EP091831 PMC12576017

[5] Charest J, Grandner MA. Sleep and athletic performance: impacts on physical performance, mental performance, injury risk and recovery, and mental health. Sleep Med Clin. 2020;15(1):41–57. 10.1016/j.jsmc.2019.11.005. 32005349 10.1016/j.jsmc.2019.11.005 PMC9960533

[6] Kline CE, Durstine JL, Davis JM, et al. Circadian variation in swim performance. J Appl Physiol. 2007;102(2):641–9. 10.1152/japplphysiol.00910.2006. 17095634 10.1152/japplphysiol.00910.2006

[7] Henst RHP, Jaspers RT, Roden LC, Rae DE. A chronotype comparison of South African and Dutch marathon runners: the role of scheduled race start times and effects on performance. Chronobiol Int. 2015;32(6):858–68. 10.3109/07420528.2015.1048870. 26102236 10.3109/07420528.2015.1048870

[8] Winter WC, Hammond WR, Green NH, Zhang Z, Bliwise DL. Measuring circadian advantage in Major League Baseball: a 10-year retrospective study. Int J Sports Physiol Perform. 2009;4(3):394–401. 10.1123/ijspp.4.3.394. 19953826 10.1123/ijspp.4.3.394

[9] Schwellnus MP, Derman WE, Jordaan E, et al. Elite athletes travelling to international destinations > 5 time zone differences from their home country have a 2–3-fold increased risk of illness. Br J Sports Med. 2012;46(11):816–21. 10.1136/bjsports-2012-091395. 22875910 10.1136/bjsports-2012-091395

[10] Janse van Rensburg DCC, Jansen van Rensburg A, Fowler P, et al. How to manage travel fatigue and jet lag in athletes? A systematic review of interventions. Br J Sports Med. 2020;54(16):960–8. 10.1136/bjsports-2019-101635. 32303523 10.1136/bjsports-2019-101635

[11] Samuels CH. Jet lag and travel fatigue: a comprehensive management plan for sport medicine physicians and high-performance support teams. Clin J Sport Med. 2012;22(3):268–73. 10.1097/JSM.0b013e31824d2eeb. 22450594 10.1097/JSM.0b013e31824d2eeb

[12] Simmons N, Mandal S, Paton B, Ahmed I. Are circadian rhythms a new frontier in athletic performance? Curr Sports Med Rep. 2022;21(1):5–7. 10.1249/JSR.0000000000000929. 35018891 10.1249/JSR.0000000000000929

[13] Blatter K, Cajochen C. Circadian rhythms in cognitive performance: methodological constraints, protocols, theoretical underpinnings. Physiol Behav. 2007;90(2–3):196–208. 10.1016/j.physbeh.2006.09.009. 17055007 10.1016/j.physbeh.2006.09.009

[14] Dallmann R, Brown SA, Gachon F. Chronopharmacology: new insights and therapeutic implications. Annu Rev Pharmacol Toxicol. 2014;54(1):339–61. 10.1146/annurev-pharmtox-011613-135923. 24160700 10.1146/annurev-pharmtox-011613-135923 PMC3885389

[15] Stephan FK. The “other” circadian system: food as a zeitgeber. J Biol Rhythms. 2002;17(4):284–92. 10.1177/074873040201700402. 12164245 10.1177/074873040201700402

[16] Roenneberg T, Kantermann T, Juda M, Vetter C, Allebrandt KV. Light and the human circadian clock. Handb Exp Pharmacol. 2013;217:311–31. 10.1007/978-3-642-25950-0_13. 10.1007/978-3-642-25950-0_13 23604485

[17] Lewis P, Korf HW, Kuffer L, Groß JV, Erren TC. Exercise time cues (zeitgebers) for human circadian systems can foster health and improve performance: a systematic review. BMJ Open Sport Exerc Med. 2018;4(1):e000443. 10.1136/bmjsem-2018-000443. 30687511 10.1136/bmjsem-2018-000443 PMC6330200

[18] Manfredini R, Manfredini F, Fersini C, Conconi F. Circadian rhythms, athletic performance, and jet lag. Br J Sports Med. 1998;32(2):101–6. 10.1136/bjsm.32.2.101. 9631214 10.1136/bjsm.32.2.101 PMC1756080

[19] Waterhouse J, Reilly T, Atkinson G. Jet-lag. Lancet. 1997;350(9091):1611–6. 10.1016/S0140-6736(97)07569-7. 9393352 10.1016/S0140-6736(97)07569-7

[20] Janse van Rensburg DC, Jansen van Rensburg A, Fowler PM, et al. Managing travel fatigue and jet lag in athletes: A review and consensus statement. Sports Med. 2021;51(10):2029–50. 10.1007/s40279-021-01502-0. 34263388 10.1007/s40279-021-01502-0 PMC8279034

[21] Takahashi T, Sasaki M, Itoh H, et al. Re-entrainment of circadian rhythm of plasma melatonin on an 8-h eastward flight. Psychiatry Clin Neurosci. 1999;53(2):257–60. 10.1046/j.1440-1819.1999.00537.x. 10459704 10.1046/j.1440-1819.1999.00537.x

[22] Leota J, Hoffman D, Czeisler MÉ, et al. Eastward jet lag is associated with impaired performance and game outcome in the National Basketball Association. Front Physiol. 2022;13:892681. 10.3389/fphys.2022.892681. 35784873 10.3389/fphys.2022.892681 PMC9245584

[23] Aschoff J, Hoffmann K, Pohl H, Wever R. Re-entrainment of circadian rhythms after phase-shifts of the Zeitgeber. Chronobiologia. 1975;2(1):23–78. 1192905

[24] Eastman CI, Burgess HJ. How to travel the world without jet lag. Sleep Med Clin. 2009;4(2):241–55. 10.1016/j.jsmc.2009.02.006. 20204161 10.1016/j.jsmc.2009.02.006 PMC2829880

[25] Silverman D, Gendreau M. Medical issues associated with commercial flights. Lancet. 2009;373(9680):2067–77. 10.1016/S0140-6736(09)60209-9. 19232708 10.1016/S0140-6736(09)60209-9 PMC7137984

[26] Lastella M, Roach GD, Sargent C. Travel fatigue and sleep/wake behaviors of professional soccer players during international competition. Sleep Health. 2019;5(2):141–7. 10.1016/j.sleh.2018.10.013. 30928113 10.1016/j.sleh.2018.10.013

[27] Fullagar HHK, Skorski S, Duffield R, Hammes D, Coutts AJ, Meyer T. Sleep and athletic performance: the effects of sleep loss on exercise performance, and physiological and cognitive responses to exercise. Sports Med. 2015;45(2):161–86. 10.1007/s40279-014-0260-0. 25315456 10.1007/s40279-014-0260-0

[28] Doherty R, Madigan SM, Nevill A, Warrington G, Ellis JG. The impact of long haul travel on the sleep of elite athletes. Neurobiol Sleep Circadian Rhythms. 2023;15(100102):100102. 10.1016/j.nbscr.2023.100102. 37766939 10.1016/j.nbscr.2023.100102 PMC10520441

[29] Biggins M, Purtill H, Fowler P, O’Sullivan K, Cahalan R. Impact of long-haul travel to international competition on sleep and recovery in elite male and female soccer athletes. Int J Sports Physiol Perform. 2022;17(9):1361–70. 10.1123/ijspp.2021-0165. 35172276 10.1123/ijspp.2021-0165

[30] Ruuskanen O, Dollner H, Luoto R, Valtonen M, Heinonen OJ, Waris M. Contraction of respiratory viral infection during air travel: an under-recognized health risk for athletes. Sports Med - Open. 2024;10(1):60. 10.1186/s40798-024-00725-5. 38776030 10.1186/s40798-024-00725-5 PMC11111432

[31] Murray HW. The pretravel consultation: recent updates. Am J Med. 2020;133(8):916-923.e2. 10.1016/j.amjmed.2020.02.005. 32179056 10.1016/j.amjmed.2020.02.005

[32] McElheny KD, Little D, Taylor D, Manzi JE. Communicable illness mitigation strategies for traveling elite sporting organizations. Sports Health. 2022;14(4):532–7. 10.1177/19417381211032226. 34292110 10.1177/19417381211032226 PMC9214894

[33] Łagowska K, Bajerska J. Probiotic supplementation and respiratory infection and immune function in athletes: systematic review and meta-analysis of randomized controlled trials. J Athl Train. 2021;56(11):1213–23. 10.4085/592-20. 33481001 10.4085/592-20 PMC8582629

[34] Besedovsky L, Lange T, Born J. Sleep and immune function. Pflugers Arch. 2012;463(1):121–37. 10.1007/s00424-011-1044-0. 22071480 10.1007/s00424-011-1044-0 PMC3256323

[35] Sopori M. Effects of cigarette smoke on the immune system. Nat Rev Immunol. 2002;2(5):372–7. 10.1038/nri803. 12033743 10.1038/nri803

[36] Green B, Bourne MN, van Dyk N, Pizzari T. Recalibrating the risk of hamstring strain injury (HSI): a 2020 systematic review and meta-analysis of risk factors for index and recurrent hamstring strain injury in sport. Br J Sports Med. 2020;54(18):1081–8. 10.1136/bjsports-2019-100983. 32299793 10.1136/bjsports-2019-100983

[37] Lisman P, Ritland BM, Burke TM, Sweeney L, Dobrosielski DA. The association between sleep and musculoskeletal injuries in military personnel: a systematic review. Mil Med. 2022;187(11–12):1318–29. 10.1093/milmed/usac118. 35544342 10.1093/milmed/usac118

[38] Milewski MD, Skaggs DL, Bishop GA, et al. Chronic lack of sleep is associated with increased sports injuries in adolescent athletes. J Pediatr Orthop. 2014;34(2):129–33. 10.1097/BPO.0000000000000151. 25028798 10.1097/BPO.0000000000000151

[39] Luke A, Lazaro RM, Bergeron MF, et al. Sports-related injuries in youth athletes: is overscheduling a risk factor? Clin J Sport Med. 2011;21(4):307–14. 10.1097/JSM.0b013e3182218f71. 21694586 10.1097/JSM.0b013e3182218f71

[40] Greenleaf JE, Farrell PA, Loomis JL, et al. Sodium chloride-citrate beverages attenuate hypovolemia in men resting 12 h at 2800 m altitude. Aviat Space Environ Med. 1998;69(10):936–43. 9773893

[41] Landgraf H, Vanselow B, Schulte-Huermann D, Mülmann MV, Bergau L. Economy class syndrome: rheology, fluid balance, and lower leg edema during a simulated 12-hour long distance flight. Aviat Space Environ Med. 1994;65(10 Pt 1):930–5. 7832736

[42] Sawka MN, Burke LM, American College of Sports Medicine, et al. American College of Sports Medicine position stand Exercise and fluid replacement. Med Sci Sports Exerc. 2007;39(2):377–90. 10.1249/mss.0b013e31802ca597. 17277604 10.1249/mss.0b013e31802ca597

[43] Savoie FA, Kenefick RW, Ely BR, Cheuvront SN, Goulet EDB. Effect of hypohydration on muscle endurance, strength, anaerobic power and capacity and vertical jumping ability: a meta-analysis. Sports Med. 2015;45(8):1207–27. 10.1007/s40279-015-0349-0. 26178327 10.1007/s40279-015-0349-0

[44] Goodman SPJ, Moreland AT, Marino FE. The effect of active hypohydration on cognitive function: a systematic review and meta-analysis. Physiol Behav. 2019;204:297–308. 10.1016/j.physbeh.2019.03.008. 30876770 10.1016/j.physbeh.2019.03.008

[45] Zubac D, Buoite Stella A, Morrison SA. Up in the air: evidence of dehydration risk and long-haul flight on athletic performance. Nutrients. 2020;12(9):2574. 10.3390/nu12092574. 32854320 10.3390/nu12092574 PMC7551461

[46] Epstein M. Alcohol’s impact on kidney function. Alcohol Health Res World. 1997;21(1):84–92. 15706766 PMC6826793

[47] Domínguez R, Veiga-Herreros P, Sánchez-Oliver AJ, et al. Acute effects of caffeine intake on psychological responses and high-intensity exercise performance. Int J Environ Res Public Health. 2021;18(2):584. 10.3390/ijerph18020584. 33445587 10.3390/ijerph18020584 PMC7827590

[48] Killer SC, Blannin AK, Jeukendrup AE. No evidence of dehydration with moderate daily coffee intake: a counterbalanced cross-over study in a free-living population. PLoS ONE. 2014;9(1):e84154. 10.1371/journal.pone.0084154. 24416202 10.1371/journal.pone.0084154 PMC3886980

[49] Patel AR, Oheb D, Zaslow TL. Gastrointestinal prophylaxis in sports medicine. Sports Health. 2018;10(2):152–5. 10.1177/194173817732733. 28952896 10.1177/1941738117732733 PMC5857727

[50] Tuteja AK, Talley NJ, Gelman SS, et al. Development of functional diarrhea, constipation, irritable bowel syndrome, and dyspepsia during and after traveling outside the USA. Dig Dis Sci. 2008;53(1):271–6. 10.1007/s10620-007-9853-x. 17549631 10.1007/s10620-007-9853-x

[51] Boggess BR. Gastrointestinal infections in the traveling athlete. Curr Sports Med Rep. 2007;6(2):125–9. 10.1007/bf02941154. 17376342 10.1007/BF02941154

[52] Fayock K, Voltz M, Sandella B, Close J, Lunser M, Okon J. Antibiotic precautions in athletes. Sports Health. 2014;6(4):321–5. 10.1177/1941738113506553. 24982704 10.1177/1941738113506553 PMC4065554

[53] Thursby E, Juge N. Introduction to the human gut microbiota. Biochem J. 2017;474(11):1823–36. 10.1042/BCJ20160510. 28512250 10.1042/BCJ20160510 PMC5433529

[54] Cook MD, Allen JM, Pence BD, et al. Exercise and gut immune function: evidence of alterations in colon immune cell homeostasis and microbiome characteristics with exercise training. Immunol Cell Biol. 2016;94(2):158–63. 10.1038/icb.2015.108. 26626721 10.1038/icb.2015.108

[55] Grosicki GJ, Durk RP, Bagley JR. Rapid gut microbiome changes in a world-class ultramarathon runner. Physiol Rep. 2019;7(24):e14313. 10.14814/phy2.14313. 31872558 10.14814/phy2.14313 PMC6928244

[56] Scheiman J, Luber JM, Chavkin TA, et al. Meta-omics analysis of elite athletes identifies a performance-enhancing microbe that functions via lactate metabolism. Nat Med. 2019;25(7):1104–9. 10.1038/s41591-019-0485-4. 31235964 10.1038/s41591-019-0485-4 PMC7368972

[57] Mohr AE, Jäger R, Carpenter KC, et al. The athletic gut microbiota. J Int Soc Sports Nutr. 2020;17(1):24. 10.1186/s12970-020-00353-w. 32398103 10.1186/s12970-020-00353-w PMC7218537

[58] O’Brien MT, O’Sullivan O, Claesson MJ, Cotter PD. The athlete gut microbiome and its relevance to health and performance: a review. Sports Med. 2022;52(Suppl 1):119–28. 10.1007/s40279-022-01785-x. 36396898 10.1007/s40279-022-01785-x PMC9734205

[59] Grosicki GJ, Pugh J, Wosinska L, et al. Ultra-endurance triathlon competition shifts fecal metabolome independent of changes to microbiome composition. J Appl Physiol. 2023;135(3):549–58. 10.1152/japplphysiol.00024.2023. 37391884 10.1152/japplphysiol.00024.2023

[60] Grosicki GJ, Langan SP, Bagley JR, et al. Gut check: unveiling the influence of acute exercise on the gut microbiota. Exp Physiol. 2023;108(12):1466–80. 10.1113/EP091446. 37702557 10.1113/EP091446 PMC10988526

[61] Baur DA, Willingham BD, Smith KA, et al. Adipose lipolysis unchanged by preexercise carbohydrate regardless of glycemic index. Med Sci Sports Exerc. 2018;50(4):827–36. 10.1249/MSS.0000000000001498. 29166321 10.1249/MSS.0000000000001498

[62] Furber MJW, Young GR, Holt GS, et al. Gut microbial stability is associated with greater endurance performance in athletes undertaking dietary periodization. mSystems. 2022;7(3):e0012922. 10.1128/msystems.00129-22. 35579384 10.1128/msystems.00129-22 PMC9238380

[63] Odonovan CM, Connor B, Madigan SM, Cotter PD, O’ Sullivan O. Instances of altered gut microbiomes among Irish cricketers over periods of travel in the lead up to the 2016 World Cup: A sequencing analysis. Travel Med Infect Dis. 2020;35:101553. 10.1016/j.tmaid.2020.101553. 31935465 10.1016/j.tmaid.2020.101553

[64] Henares D, Monsalvez V, Brotons P, et al. Human gut microbiota composition associated with international travels. Travel Med Infect Dis. 2024;61(102747):102747. 10.1016/j.tmaid.2024.102747. 39094984 10.1016/j.tmaid.2024.102747

[65] Kampmann C, Dicksved J, Engstrand L, Rautelin H. Changes to human faecal microbiota after international travel. Travel Med Infect Dis. 2021;44(102199):102199. 10.1016/j.tmaid.2021.102199. 34781018 10.1016/j.tmaid.2021.102199

[66] Youmans BP, Ajami NJ, Jiang ZD, et al. Characterization of the human gut microbiome during travelers’ diarrhea. Gut Microbes. 2015;6(2):110–9. 10.1080/19490976.2015.1019693. 25695334 10.1080/19490976.2015.1019693 PMC4615231

[67] Mashaqi S, Gozal D. “Circadian misalignment and the gut microbiome. A bidirectional relationship triggering inflammation and metabolic disorders”- a literature review. Sleep Med. 2020;72:93–108. 10.1016/j.sleep.2020.03.020. 32559717 10.1016/j.sleep.2020.03.020

[68] Yamaoka K, Uotsu N, Hoshino E. Relationship between psychosocial stress-induced prefrontal cortex activity and gut microbiota in healthy participants-a functional near-infrared spectroscopy study. Neurobiol Stress. 2022;20(100479):100479. 10.1016/j.ynstr.2022.100479. 36039149 10.1016/j.ynstr.2022.100479 PMC9418982

[69] Jäger R, Mohr AE, Carpenter KC, et al. International Society of Sports Nutrition position stand: probiotics. J Int Soc Sports Nutr. 2019;16(1):62. 10.1186/s12970-019-0329-0. 31864419 10.1186/s12970-019-0329-0 PMC6925426

[70] Mohr AE, Basile AJ, Crawford MS, Sweazea KL, Carpenter KC. Probiotic supplementation has a limited effect on circulating immune and inflammatory markers in healthy adults: a systematic review of randomized controlled trials. J Acad Nutr Diet. 2020;120(4):548–64. 10.1016/j.jand.2019.08.018. 31648930 10.1016/j.jand.2019.08.018

[71] Pugh JN, Sparks AS, Doran DA, et al. Four weeks of probiotic supplementation reduces GI symptoms during a marathon race. Eur J Appl Physiol. 2019;119(7):1491–501. 10.1007/s00421-019-04136-3. 30982100 10.1007/s00421-019-04136-3 PMC6570661

[72] Smith RS, Efron B, Mah CD, Malhotra A. The impact of circadian misalignment on athletic performance in professional football players. Sleep. 2013;36(12):1999–2001. 10.5665/sleep.3248. 24293776 10.5665/sleep.3248 PMC3825451

[73] Chellappa SL, Morris CJ, Scheer FAJL. Daily circadian misalignment impairs human cognitive performance task-dependently. Sci Rep. 2018;8(1):3041. 10.1038/s41598-018-20707-4. 29445188 10.1038/s41598-018-20707-4 PMC5812992

[74] Charest J, Cook JD, Bender AM, Walch O, Grandner MA, Samuels CH. Associations between time zone changes, travel distance and performance: a retrospective analysis of 2013-2020 National Hockey League data. J Sci Med Sport. 2022;25(12):1008–16. 10.1016/j.jsams.2022.10.005. 36319561 10.1016/j.jsams.2022.10.005

[75] Botonis PG, Toubekis AG, Hill DW. Effects of consecutive long-haul travel on jet-lag symptoms: case study of a national water polo team preparing for world-class events. Int J Sports Physiol Perform. 2026;21(2):267–73. 10.1123/ijspp.2025-0258. 41468192 10.1123/ijspp.2025-0258

[76] Frisco DJ, Goodrich JA, Byrnes WC, Holliday M, Wright KP. 0228 the impact of westward travel across 9 time zones on sleep behaviors of female collegiate athletes. Sleep. 2020;43(Supplement_1):A88–A88. 10.1093/sleep/zsaa056.226.

[77] Lemmer B, Kern RI, Nold G, Lohrer H. Jet lag in athletes after eastward and westward time-zone transition. Chronobiol Int. 2002;19(4):743–64. 10.1081/cbi-120005391. 12182501 10.1081/cbi-120005391

[78] Song A, Severini T, Allada R. How jet lag impairs Major League Baseball performance. Proc Natl Acad Sci U S A. 2017;114(6):1407–12. 10.1073/pnas.1608847114. 28115724 10.1073/pnas.1608847114 PMC5307448

[79] Recht LD, Lew RA, Schwartz WJ. Baseball teams beaten by jet lag. Nature. 1995;377(6550):583. 10.1038/377583a0. 7566168 10.1038/377583a0

[80] Glinski J, Chandy D. Impact of jet lag on free throw shooting in the National Basketball Association. Chronobiol Int. 2022;39(7):1001–5. 10.1080/07420528.2022.2057321. 35345951 10.1080/07420528.2022.2057321

[81] Jehue R, Street D, Huizenga R. Effect of time zone and game time changes on team performance: National Football League. Med Sci Sports Exerc. 1993;25(1):127–31. 10.1249/00005768-199301000-00017. 8423745 10.1249/00005768-199301000-00017

[82] Lok R, Zerbini G, Gordijn MCM, Beersma DGM, Hut RA. Gold, silver or bronze: circadian variation strongly affects performance in Olympic athletes. Sci Rep. 2020;10(1):16088. 10.1038/s41598-020-72573-8. 33033271 10.1038/s41598-020-72573-8 PMC7544825

[83] Craven J, McCartney D, Desbrow B, et al. Effects of acute sleep loss on physical performance: A systematic and meta-analytical review. Sports Med. 2022;52(11):2669–90. 10.1007/s40279-022-01706-y. 35708888 10.1007/s40279-022-01706-y PMC9584849

[84] Chapman DW, Bullock N, Ross A, Rosemond D, Martin DT. Detrimental effects of west to east transmeridian flight on jump performance. Eur J Appl Physiol. 2012;112(5):1663–9. 10.1007/s00421-011-2134-6. 21874551 10.1007/s00421-011-2134-6

[85] Zeitzer JM, Dijk DJ, Kronauer R, Brown E, Czeisler C. Sensitivity of the human circadian pacemaker to nocturnal light: melatonin phase resetting and suppression. J Physiol. 2000;526(Pt 3):695–702. 10.1111/j.1469-7793.2000.00695.x. 10922269 10.1111/j.1469-7793.2000.00695.x PMC2270041

[86] Khalsa SBS, Jewett ME, Cajochen C, Czeisler CA. A phase response curve to single bright light pulses in human subjects. J Physiol. 2003;549(Pt 3):945–52. 10.1113/jphysiol.2003.040477. 12717008 10.1113/jphysiol.2003.040477 PMC2342968

[87] Horne JA, Ostberg O. A self-assessment questionnaire to determine morningness-eveningness in human circadian rhythms. Int J Chronobiol. 1976;4(2):97–110. 1027738

[88] Roenneberg T, Wirz-Justice A, Merrow M. Life between clocks: daily temporal patterns of human chronotypes. J Biol Rhythms. 2003;18(1):80–90. 10.1177/0748730402239679. 12568247 10.1177/0748730402239679

[89] Montaruli A, Castelli L, Mulè A, et al. Biological rhythm and chronotype: New perspectives in health. Biomolecules. 2021;11(4):487. 10.3390/biom11040487. 33804974 10.3390/biom11040487 PMC8063933

[90] Facer-Childs E, Brandstaetter R. The impact of circadian phenotype and time since awakening on diurnal performance in athletes. Curr Biol. 2015;25(4):518–22. 10.1016/j.cub.2014.12.036. 25639241 10.1016/j.cub.2014.12.036

[91] Ghotbi N, Pilz LK, Winnebeck EC, et al. The µMCTQ: An ultra-short version of the Munich ChronoType Questionnaire. J Biol Rhythms. 2020;35(1):98–110. 10.1177/0748730419886986. 31791166 10.1177/0748730419886986

[92] Kantermann T, Sung H, Burgess HJ. Comparing the Morningness-Eveningness Questionnaire and Munich ChronoType Questionnaire to the dim light melatonin onset. J Biol Rhythms. 2015;30(5):449–53. 10.1177/0748730415597520. 26243627 10.1177/0748730415597520 PMC4580371

[93] Adan A, Almirall H. Horne & Östberg morningness-eveningness questionnaire: A reduced scale. Pers Individ Dif. 1991;12(3):241–53. 10.1016/0191-8869(91)90110-w.

[94] Facer-Childs E, Brandstaetter R. Circadian phenotype composition is a major predictor of diurnal physical performance in teams. Front Neurol. 2015;6:208. 10.3389/fneur.2015.00208. 26483754 10.3389/fneur.2015.00208 PMC4589674

[95] Waterhouse J, Edwards B, Nevill A, et al. Identifying some determinants of “jet lag” and its symptoms: a study of athletes and other travellers. Br J Sports Med. 2002;36(1):54–60. 10.1136/bjsm.36.1.54. 11867494 10.1136/bjsm.36.1.54 PMC1724441

[96] Wittmann M, Dinich J, Merrow M, Roenneberg T. Social jetlag: misalignment of biological and social time. Chronobiol Int. 2006;23(1–2):497–509. 10.1080/07420520500545979. 16687322 10.1080/07420520500545979

[97] Facer-Childs ER, Boiling S, Balanos GM. The effects of time of day and chronotype on cognitive and physical performance in healthy volunteers. Sports Med Open. 2018;4(1):47. 10.1186/s40798-018-0162-z. 30357501 10.1186/s40798-018-0162-z PMC6200828

[98] Maheshwari D, Singla D, Malhotra D, Zutshi K. Circadian chronotypes and their effect on athletic performance: a systematic review. Sport Sci Health. 2022;18(4):1161–77. 10.1007/s11332-022-00929-w.

[99] Nedelec M, Aloulou A, Duforez F, Meyer T, Dupont G. The variability of sleep among elite athletes. Sports Med Open. 2018;4(1):34. 10.1186/s40798-018-0151-2. 30054756 10.1186/s40798-018-0151-2 PMC6063976

[100] Phillips AJK, Clerx WM, O’Brien CS, et al. Irregular sleep/wake patterns are associated with poorer academic performance and delayed circadian and sleep/wake timing. Sci Rep. 2017;7(1):3216. 10.1038/s41598-017-03171-4. 28607474 10.1038/s41598-017-03171-4 PMC5468315

[101] Varesco G, Yao CW, Dubé E, Simonelli G, Bieuzen F. The impact of long-haul travel and 13 h time change on sleep and rest activity circadian rhythm in speed skaters during World Cup competitions. Exp Physiol. 2025;110(11):1732–43. 10.1113/EP092195. 39487563 10.1113/EP092195 PMC12576015

[102] Lemola S, Ledermann T, Friedman EM. Variability of sleep duration is related to subjective sleep quality and subjective well-being: an actigraphy study. PLoS ONE. 2013;8(8):e71292. 10.1371/journal.pone.0071292. 23967186 10.1371/journal.pone.0071292 PMC3743871

[103] Bei B, Wiley JF, Trinder J, Manber R. Beyond the mean: a systematic review on the correlates of daily intraindividual variability of sleep/wake patterns. Sleep Med Rev. 2016;28:108–24. 10.1016/j.smrv.2015.06.003. 26588182 10.1016/j.smrv.2015.06.003

[104] Grosicki GJ, von Hippel W, Fielding F, Kim J, Chapman C, Holmes KE. Wearable-derived sleep and physiological metrics are associated with performance in professional golfers. Int J Sports Physiol Perform. 2026;21(2):180–91. 10.1123/ijspp.2025-0226. 41265431 10.1123/ijspp.2025-0226

[105] Mah CD, Mah KE, Kezirian EJ, Dement WC. The effects of sleep extension on the athletic performance of collegiate basketball players. Sleep. 2011;34(7):943–50. 10.5665/SLEEP.1132. 21731144 10.5665/SLEEP.1132 PMC3119836

[106] Bonnar D, Bartel K, Kakoschke N, Lang C. Sleep interventions designed to improve athletic performance and recovery: a systematic review of current approaches. Sports Med. 2018;48(3):683–703. 10.1007/s40279-017-0832-x. 29352373 10.1007/s40279-017-0832-x

[107] Gong M, Sun M, Sun Y, Jin L, Li S. Effects of acute sleep deprivation on sporting performance in athletes: a comprehensive systematic review and meta-analysis. Nat Sci Sleep. 2024;16:935–48. 10.2147/NSS.S467531. 39006249 10.2147/NSS.S467531 PMC11246080

[108] VanHelder T, Radomski MW. Sleep deprivation and the effect on exercise Performance1. Sports Med. 1989;7(4):235–47. 10.2165/00007256-198907040-00002. 2657963 10.2165/00007256-198907040-00002

[109] Souissi M, Souissi Y, Bayoudh A, Knechtle B, Nikolaidis PT, Chtourou H. Effects of a 30 min nap opportunity on cognitive and short-duration high-intensity performances and mood states after a partial sleep deprivation night. J Sports Sci. 2020;38(22):2553–61. 10.1080/02640414.2020.1793651. 32734824 10.1080/02640414.2020.1793651

[110] Abdessalem R, Boukhris O, Hsouna H, et al. Effect of napping opportunity at different times of day on vigilance and shuttle run performance. Chronobiol Int. 2019;36(10):1334–42. 10.1080/07420528.2019.1642908. 31368367 10.1080/07420528.2019.1642908

[111] Boukhris O, Trabelsi K, Ammar A, et al. A 90 min daytime nap opportunity is better than 40 min for cognitive and physical performance. Int J Environ Res Public Health. 2020;17(13):4650. 10.3390/ijerph17134650. 32605240 10.3390/ijerph17134650 PMC7369743

[112] Thun E, Bjorvatn B, Flo E, Harris A, Pallesen S. Sleep, circadian rhythms, and athletic performance. Sleep Med Rev. 2015;23:1–9. 10.1016/j.smrv.2014.11.003. 25645125 10.1016/j.smrv.2014.11.003

[113] Lastella M, Halson SL, Vitale JA, Memon AR, Vincent GE. To nap or not to nap? A systematic review evaluating napping behavior in athletes and the impact on various measures of athletic performance. Nat Sci Sleep. 2021;13:841–62. 10.2147/NSS.S315556. 34194254 10.2147/NSS.S315556 PMC8238550

[114] Botonis PG, Koutouvakis N, Toubekis AG. The impact of daytime napping on athletic performance - a narrative review. Scand J Med Sci Sports. 2021;31(12):2164–77. 10.1111/sms.14060. 34559915 10.1111/sms.14060

[115] Boivin DB, Duffy JF, Kronauer RE, Czeisler CA. Dose-response relationships for resetting of human circadian clock by light. Nature. 1996;379(6565):540–2. 10.1038/379540a0. 8596632 10.1038/379540a0

[116] Czeisler CA, Duffy JF, Shanahan TL, et al. Stability, precision, and near-24-hour period of the human circadian pacemaker. Science. 1999;284(5423):2177–81. 10.1126/science.284.5423.2177. 10381883 10.1126/science.284.5423.2177

[117] Reppert SM, Weaver DR. Coordination of circadian timing in mammals. Nature. 2002;418(6901):935–41. 10.1038/nature00965. 12198538 10.1038/nature00965

[118] Schmidt C, Collette F, Cajochen C, Peigneux P. A time to think: circadian rhythms in human cognition. Cogn Neuropsychol. 2007;24(7):755–89. 10.1080/02643290701754158. 18066734 10.1080/02643290701754158

[119] Bano-Otalora B, Martial F, Harding C, et al. Bright daytime light enhances circadian amplitude in a diurnal mammal. Proc Natl Acad Sci U S A. 2021;118(22):e2100094118. 10.1073/pnas.2100094118. 34031246 10.1073/pnas.2100094118 PMC8179182

[120] Arendt J. Managing jet lag: some of the problems and possible new solutions. Sleep Med Rev. 2009;13(4):249–56. 10.1016/j.smrv.2008.07.011. 19147377 10.1016/j.smrv.2008.07.011

[121] Bin YS, Postnova S, Cistulli PA. What works for jetlag? A systematic review of non-pharmacological interventions. Sleep Med Rev. 2019;43:47–59. 10.1016/j.smrv.2018.09.005. 30529430 10.1016/j.smrv.2018.09.005

[122] Gooley JJ, Rajaratnam SMW, Brainard GC, Kronauer RE, Czeisler CA, Lockley SW. Spectral responses of the human circadian system depend on the irradiance and duration of exposure to light. Sci Transl Med. 2010;2(31):31ra33. 10.1126/scitranslmed.3000741. 20463367 10.1126/scitranslmed.3000741 PMC4414925

[123] Rüger M, St Hilaire MA, Brainard GC, et al. Human phase response curve to a single 6.5 h pulse of short-wavelength light. J Physiol. 2013;591(1):353–63. 10.1113/jphysiol.2012.239046. 23090946 10.1113/jphysiol.2012.239046 PMC3630790

[124] St Hilaire MA, Gooley JJ, Khalsa SBS, Kronauer RE, Czeisler CA, Lockley SW. Human phase response curve to a 1 h pulse of bright white light. J Physiol. 2012;590(13):3035–45. 10.1113/jphysiol.2012.227892. 22547633 10.1113/jphysiol.2012.227892 PMC3406389

[125] Kripke DF, Elliott JA, Youngstedt SD, Rex KM. Circadian phase response curves to light in older and young women and men. J Circadian Rhythms. 2007;5(0):4. 10.1186/1740-3391-5-4. 17623102 10.1186/1740-3391-5-4 PMC1988787

[126] Youngstedt SD, Kline CE, Elliott JA, Zielinski MR, Devlin TM, Moore TA. Circadian phase-shifting effects of bright light, exercise, and bright light + exercise. J Circadian Rhythms. 2016;14(1):2. 10.5334/jcr.137. 27103935 10.5334/jcr.137 PMC4834751

[127] Barger LK, Wright KP Jr, Hughes RJ, Czeisler CA. Daily exercise facilitates phase delays of circadian melatonin rhythm in very dim light. Am J Physiol Regul Integr Comp Physiol. 2004;286(6):R1077–84. 10.1152/ajpregu.00397.2003. 15031136 10.1152/ajpregu.00397.2003

[128] Buxton OM, Lee CW, L’Hermite-Baleriaux M, Turek FW, Van Cauter E. Exercise elicits phase shifts and acute alterations of melatonin that vary with circadian phase. Am J Physiol Regul Integr Comp Physiol. 2003;284(3):R714–24. 10.1152/ajpregu.00355.2002. 12571075 10.1152/ajpregu.00355.2002

[129] Leota J, Presby DM, Le F, et al. Dose-response relationship between evening exercise and sleep. Nat Commun. 2025;16(1):3297. 10.1038/s41467-025-58271-x. 40234380 10.1038/s41467-025-58271-x PMC12000559

[130] Bytomski JR. Fueling for performance. Sports Health. 2018;10(1):47–53. 10.1177/1941738117743913. 29173121 10.1177/1941738117743913 PMC5753973

[131] Nolte J, Kirmse M, de Marées M, Platen P. Effects of short-term low energy availability on metabolism and performance-related parameters in physically active adults. Nutrients. 2025. 10.3390/nu17020278. 39861408 10.3390/nu17020278 PMC11767613

[132] Kojima C, Ishibashi A, Tanabe Y, et al. Muscle glycogen content during endurance training under low energy availability. Med Sci Sports Exerc. 2020;52(1):187–95. 10.1249/MSS.0000000000002098. 31343520 10.1249/MSS.0000000000002098

[133] Jeppesen JS, Caldwell HG, Lossius LO, et al. Low energy availability increases immune cell formation of reactive oxygen species and impairs exercise performance in female endurance athletes. Redox Biol. 2024;75(103250):103250. 10.1016/j.redox.2024.103250. 38936255 10.1016/j.redox.2024.103250 PMC11260862

[134] Oxfeldt M, Phillips SM, Andersen OE, et al. Low energy availability reduces myofibrillar and sarcoplasmic muscle protein synthesis in trained females. J Physiol. 2023;601(16):3481–97. 10.1113/JP284967. 37329147 10.1113/JP284967

[135] Zachwieja JJ, Ezell DM, Cline AD, et al. Short-term dietary energy restriction reduces lean body mass but not performance in physically active men and women. Int J Sports Med. 2001;22(4):310–6. 10.1055/s-2001-13822. 11414677 10.1055/s-2001-13822

[136] Gallant TL, Ong LF, Wong L, et al. Low energy availability and relative energy deficiency in sport: a systematic review and meta-analysis. Sports Med. 2025;55(2):325–39. 10.1007/s40279-024-02130-0. 39485653 10.1007/s40279-024-02130-0

[137] Taleb Z, Karpowicz P. Circadian regulation of digestive and metabolic tissues. Am J Physiol Cell Physiol. 2022;323(2):C306–21. 10.1152/ajpcell.00166.2022. 35675638 10.1152/ajpcell.00166.2022

[138] Duboc H, Coffin B, Siproudhis L. Disruption of circadian rhythms and gut motility: an overview of underlying mechanisms and associated pathologies. J Clin Gastroenterol. 2020;54(5):405–14. 10.1097/MCG.0000000000001333. 32134798 10.1097/MCG.0000000000001333 PMC7147411

[139] Halson SL, Burke LM, Pearce J. Nutrition for travel: from jet lag to catering. Int J Sport Nutr Exerc Metab. 2019;29(2):228–35. 10.1123/ijsnem.2018-0278. 30507257 10.1123/ijsnem.2018-0278

[140] Potter GDM, Cade JE, Grant PJ, Hardie LJ. Nutrition and the circadian system. Br J Nutr. 2016;116(3):434–42. 10.1017/S0007114516002117. 27221157 10.1017/S0007114516002117 PMC4930144

[141] Lamia KA, Storch KF, Weitz CJ. Physiological significance of a peripheral tissue circadian clock. Proc Natl Acad Sci U S A. 2008;105(39):15172–7. 10.1073/pnas.0806717105. 18779586 10.1073/pnas.0806717105 PMC2532700

[142] Wehrens SMT, Christou S, Isherwood C, et al. Meal timing regulates the human circadian system. Curr Biol. 2017;27(12):1768-1775.e3. 10.1016/j.cub.2017.04.059. 28578930 10.1016/j.cub.2017.04.059 PMC5483233

[143] Halson SL. Sleep in elite athletes and nutritional interventions to enhance sleep. Sports Med. 2014;44(Suppl 1(S1)):S13–23. 10.1007/s40279-014-0147-0. 24791913 10.1007/s40279-014-0147-0 PMC4008810

[144] Daniel PM, Love ER, Moorhouse SR, Pratt OE. The effect of insulin upon the influx of tryptophan into the brain of the rabbit. J Physiol. 1981;312(1):551–62. 10.1113/jphysiol.1981.sp013643. 7021801 10.1113/jphysiol.1981.sp013643 PMC1275568

[145] Afaghi A, O’Connor H, Chow CM. High-glycemic-index carbohydrate meals shorten sleep onset. Am J Clin Nutr. 2007;85(2):426–30. 10.1093/ajcn/85.2.426. 17284739 10.1093/ajcn/85.2.426

[146] Vlahoyiannis A, Giannaki CD, Sakkas GK, Aphamis G, Andreou E. A systematic review, meta-analysis and meta-regression on the effects of carbohydrates on sleep. Nutrients. 2021;13(4):1283. 10.3390/nu13041283. 33919698 10.3390/nu13041283 PMC8069918

[147] Afaghi A, O’Connor H, Chow CM. Acute effects of the very low carbohydrate diet on sleep indices. Nutr Neurosci. 2008;11(4):146–54. 10.1179/147683008X301540. 18681982 10.1179/147683008X301540

[148] Cowan AE, Tooze JA, Gahche JJ, et al. Trends in overall and micronutrient-containing dietary supplement use in US adults and children, NHANES 2007–2018. J Nutr. 2023;152(12):2789–801. 10.1093/jn/nxac168. 35918260 10.1093/jn/nxac168 PMC9839985

[149] Hurst P, Kavussanu M, Davies R, Dallaway N, Ring C. Use of sport supplements and doping substances by athletes: prevalence and relationships. J Clin Med. 2024. 10.3390/jcm13237132. 39685590 10.3390/jcm13237132 PMC11642664

[150] Chan V, Wang L, Allman-Farinelli M. Efficacy of functional foods, beverages, and supplements claiming to alleviate air travel symptoms: systematic review and meta-analysis. Nutrients. 2021;13(3):961. 10.3390/nu13030961. 33809656 10.3390/nu13030961 PMC8002180

[151] Acheson KJ, Gremaud G, Meirim I, et al. Metabolic effects of caffeine in humans: lipid oxidation or futile cycling? Am J Clin Nutr. 2004;79(1):40–6. 10.1093/ajcn/79.1.40. 14684395 10.1093/ajcn/79.1.40

[152] Goldstein ER, Ziegenfuss T, Kalman D, et al. International Society of Sports Nutrition position stand: caffeine and performance. J Int Soc Sports Nutr. 2010;7(1):5. 10.1186/1550-2783-7-5. 20205813 10.1186/1550-2783-7-5 PMC2824625

[153] Forbes-Robertson S, Dudley E, Vadgama P, Cook C, Drawer S, Kilduff L. Circadian disruption and remedial interventions: effects and interventions for jet lag for athletic peak performance. Sports Med. 2012;42(3):185–208. 10.2165/11596850-000000000-00000. 22299812 10.2165/11596850-000000000-00000

[154] Tsai MT, Shiu YJ, Ho CC, Chen CH, Chiu CH. Effects of caffeinated chewing gum on ice hockey performance after jet lag intervention: double-blind crossover trial. Nutrients. 2024. 10.3390/nu16183151. 39339752 10.3390/nu16183151 PMC11434913

[155] Antonio J, Newmire DE, Stout JR, et al. Common questions and misconceptions about caffeine supplementation: what does the scientific evidence really show? J Int Soc Sports Nutr. 2024;21(1):2323919. 10.1080/15502783.2024.2323919. 38466174 10.1080/15502783.2024.2323919 PMC10930107

[156] Guest NS, VanDusseldorp TA, Nelson MT, et al. International society of sports nutrition position stand: caffeine and exercise performance. J Int Soc Sports Nutr. 2021;18(1):1. 33388079 10.1186/s12970-020-00383-4 PMC7777221

[157] Drake C, Roehrs T, Shambroom J, Roth T. Caffeine effects on sleep taken 0, 3, or 6 hours before going to bed. J Clin Sleep Med. 2013;9(11):1195–200. 10.5664/jcsm.3170. 24235903 10.5664/jcsm.3170 PMC3805807

[158] Gardiner CL, Weakley J, Burke LM, et al. Dose and timing effects of caffeine on subsequent sleep: a randomized clinical crossover trial. Sleep. 2025. 10.1093/sleep/zsae230. 39377163 10.1093/sleep/zsae230 PMC11985402

[159] Burke TM, Markwald RR, McHill AW, et al. Effects of caffeine on the human circadian clock in vivo and in vitro. Sci Transl Med. 2015;7(305):305ra146. 10.1126/scitranslmed.aac5125. 26378246 10.1126/scitranslmed.aac5125 PMC4657156

[160] Rackard G, Madigan SM, Connolly J, Keaver L, Ryan L, Doherty R. Nutrition strategies to promote sleep in elite athletes: a scoping review. Sports. 2025;13(10):342. 10.3390/sports13100342. 41150477 10.3390/sports13100342 PMC12567717

[161] Chung J, Choi M, Lee K. Effects of short-term intake of Montmorency tart cherry juice on sleep quality after intermittent exercise in elite female field hockey players: a randomized controlled trial. Int J Environ Res Public Health. 2022;19(16):10272. 10.3390/ijerph191610272. 36011907 10.3390/ijerph191610272 PMC9408103

[162] Al Alawi AM, Al Badi A, Al Huraizi A, Falhammar H. Magnesium: the recent research and developments. Adv Food Nutr Res. 2021;96:193–218. 10.1016/bs.afnr.2021.01.001. 34112353 10.1016/bs.afnr.2021.01.001

[163] Volpe SL. Magnesium and the athlete. Curr Sports Med Rep. 2015;14(4):279–83. 10.1249/JSR.0000000000000178. 26166051 10.1249/JSR.0000000000000178

[164] Pollock N, Chakraverty R, Taylor I, Killer SC. An 8-year analysis of magnesium status in elite international track & field athletes. J Am Coll Nutr. 2020;39(5):443–9. 10.1080/07315724.2019.1691953. 31829845 10.1080/07315724.2019.1691953

[165] Billyard AJ, Eggett DL, Franz KB. Dietary magnesium deficiency decreases plasma melatonin in rats. Magnes Res. 2006;19(3):157–61. 17172005

[166] Cuciureanu MD, Vink R. Magnesium and stress. In: Magnesium in the central nervous system. University of Adelaide Press; 2011. 29920004

[167] Arab A, Rafie N, Amani R, Shirani F. The role of magnesium in sleep health: a systematic review of available literature. Biol Trace Elem Res. 2023;201(1):121–8. 10.1007/s12011-022-03162-1. 35184264 10.1007/s12011-022-03162-1

[168] Rawji A, Peltier MR, Mourtzanakis K, et al. Examining the effects of supplemental magnesium on self-reported anxiety and sleep quality: a systematic review. Cureus. 2024;16(4):e59317. 10.7759/cureus.59317. 38817505 10.7759/cureus.59317 PMC11136869

[169] Slutsky I, Abumaria N, Wu LJ, et al. Enhancement of learning and memory by elevating brain magnesium. Neuron. 2010;65(2):165–77. 10.1016/j.neuron.2009.12.026. 20152124 10.1016/j.neuron.2009.12.026

[170] Hausenblas HA, Lynch T, Hooper S, Shrestha A, Rosendale D, Gu J. Magnesium-L-threonate improves sleep quality and daytime functioning in adults with self-reported sleep problems: a randomized controlled trial. Sleep Med X. 2024;8(100121):100121. 10.1016/j.sleepx.2024.100121. 39252819 10.1016/j.sleepx.2024.100121 PMC11381753

[171] Zhang C, Hu Q, Li S, et al. A Magtein®, magnesium L-threonate, -based formula improves brain cognitive functions in healthy Chinese adults. Nutrients. 2022;14(24):5235. 10.3390/nu14245235. 36558392 10.3390/nu14245235 PMC9786204

[172] Poza JJ, Pujol M, Ortega-Albás JJ, Romero O, Insomnia Study Group of the Spanish Sleep Society (SES). Melatonin in sleep disorders. Neurología (English Edition). 2022;37(7):575–85. 10.1016/j.nrleng.2018.08.004. 10.1016/j.nrleng.2018.08.004 36064286

[173] Sack RL. The pathophysiology of jet lag. Travel Med Infect Dis. 2009;7(2):102–10. 10.1016/j.tmaid.2009.01.006. 19237143 10.1016/j.tmaid.2009.01.006

[174] Ferracioli-Oda E, Qawasmi A, Bloch MH. Meta-analysis: melatonin for the treatment of primary sleep disorders. PLoS ONE. 2013;8(5):e63773. 10.1371/journal.pone.0063773. 23691095 10.1371/journal.pone.0063773 PMC3656905

[175] Choi K, Lee YJ, Park S, Je NK, Suh HS. Efficacy of melatonin for chronic insomnia: Systematic reviews and meta-analyses. Sleep Med Rev. 2022;66(101692):101692. 10.1016/j.smrv.2022.101692. 36179487 10.1016/j.smrv.2022.101692

[176] Salanitro M, Wrigley T, Ghabra H, et al. Efficacy on sleep parameters and tolerability of melatonin in individuals with sleep or mental disorders: A systematic review and meta-analysis. Neurosci Biobehav Rev. 2022;139(104723):104723. 10.1016/j.neubiorev.2022.104723. 35691474 10.1016/j.neubiorev.2022.104723

[177] Rogers NL, Kennaway DJ, Dawson D. Neurobehavioural performance effects of daytime melatonin and temazepam administration. J Sleep Res. 2003;12(3):207–12. 10.1046/j.1365-2869.2003.00360.x. 12941059 10.1046/j.1365-2869.2003.00360.x

[178] Zhdanova IV, Wurtman RJ, Morabito C, Piotrovska VR, Lynch HJ. Effects of low oral doses of melatonin, given 2-4 hours before habitual bedtime, on sleep in normal young humans. Sleep. 1996;19(5):423–31. 10.1093/sleep/19.5.423. 8843534 10.1093/sleep/19.5.423

[179] Petrie K, Dawson AG, Thompson L, Brook R. A double-blind trial of melatonin as a treatment for jet lag in international cabin crew. Biol Psychiatry. 1993;33(7):526–30. 10.1016/0006-3223(93)90007-z. 8513037 10.1016/0006-3223(93)90007-z

[180] Celorrio San Miguel AM, Roche E, Herranz-López M, Celorrio San Miguel M, Mielgo-Ayuso J, Fernández-Lázaro D. Impact of melatonin supplementation on sports performance and circulating biomarkers in highly trained athletes: a systematic review of randomized controlled trials. Nutrients. 2024. 10.3390/nu16071011. 38613044 10.3390/nu16071011 PMC11013451

[181] Sharkey KM, Eastman CI. Melatonin phase shifts human circadian rhythms in a placebo-controlled simulated night-work study. Am J Physiol Regul Integr Comp Physiol. 2002;282(2):R454–63. 10.1152/ajpregu.00135.2001. 11792655 10.1152/ajpregu.00135.2001 PMC3696986

[182] Gooneratne NS, Edwards AYZ, Zhou C, Cuellar N, Grandner MA, Barrett JS. Melatonin pharmacokinetics following two different oral surge-sustained release doses in older adults. J Pineal Res. 2012;52(4):437–45. 10.1111/j.1600-079X.2011.00958.x. 22348451 10.1111/j.1600-079X.2011.00958.x PMC3682489

[183] Zhdanova IV, Tucci V. Melatonin, circadian rhythms, and sleep. Curr Treat Options Neurol. 2003;5(3):225–9. 10.1007/s11940-003-0013-0. 12670411 10.1007/s11940-003-0013-0

[184] Khodaee M, Grothe HL, Seyfert JH, VanBaak K. Athletes at high altitude. Sports Health. 2016;8(2):126–32. 10.1177/1941738116630948. 26863894 10.1177/1941738116630948 PMC4789936

[185] Tsunekawa K, Ushiki K, Martha L, et al. Differences in stress response between two altitudes assessed by salivary cortisol levels within circadian rhythms in long-distance runners. Sci Rep. 2022;12(1):9749. 10.1038/s41598-022-13965-w. 35697776 10.1038/s41598-022-13965-w PMC9192635

[186] Garvican-Lewis LA, Halliday I, Abbiss CR, Saunders PU, Gore CJ. Altitude exposure at 1800 m increases haemoglobin mass in distance runners. J Sports Sci Med. 2015;14(2):413–7. 25983592 PMC4424472

[187] Siebenmann C, Lundby C. Regulation of cardiac output in hypoxia. Scand J Med Sci Sports. 2015;25(Suppl 4(S4)):53–9. 10.1111/sms.12619. 26589118 10.1111/sms.12619

[188] Muhm JM, Rock PB, McMullin DL, et al. Effect of aircraft-cabin altitude on passenger discomfort. N Engl J Med. 2007;357(1):18–27. 10.1056/NEJMoa062770. 17611205 10.1056/NEJMoa062770

[189] Koehle MS, Cheng I, Sporer B. Canadian Academy of Sport and Exercise Medicine position statement: athletes at high altitude. Clin J Sport Med. 2014;24(2):120–7. 10.1097/JSM.0000000000000024. 24569430 10.1097/JSM.0000000000000024

[190] Tseng CH, Lin FC, Chao HS, Tsai HC, Shiao GM, Chang SC. Impact of rapid ascent to high altitude on sleep. Sleep Breath. 2015;19(3):819–26. 10.1007/s11325-014-1093-7. 25491080 10.1007/s11325-014-1093-7

[191] Weil JV. Sleep at high altitude. High Alt Med Biol. 2004;5(2):180–9. 10.1089/1527029041352162. 15265339 10.1089/1527029041352162

[192] Kechijian D. Optimizing nutrition for performance at altitude: a literature review. J Spec Oper Med. 2011;11(1):12–7. 10.55460/LXQK-O2RD. 21455904 10.55460/LXQK-O2RD

[193] Chapman RF, Laymon Stickford AS, Lundby C, Levine BD. Timing of return from altitude training for optimal sea level performance. J Appl Physiol. 2014;116(7):837–43. 10.1152/japplphysiol.00663.2013. 24336885 10.1152/japplphysiol.00663.2013

[194] Lundby C, Millet GP, Calbet JA, Bärtsch P, Subudhi AW. Does “altitude training” increase exercise performance in elite athletes? Br J Sports Med. 2012;46(11):792–5. 10.1136/bjsports-2012-091231. 22797528 10.1136/bjsports-2012-091231

[195] Czuba M, Waskiewicz Z, Zajac A, Poprzecki S, Cholewa J, Roczniok R. The effects of intermittent hypoxic training on aerobic capacity and endurance performance in cyclists. J Sports Sci Med. 2011;10(1):175–83. 24149312 PMC3737917

[196] Périard JD, Eijsvogels TMH, Daanen HAM. Exercise under heat stress: thermoregulation, hydration, performance implications, and mitigation strategies. Physiol Rev. 2021;101(4):1873–979. 10.1152/physrev.00038.2020. 33829868 10.1152/physrev.00038.2020

[197] Millet GP, Roels B, Schmitt L, Woorons X, Richalet JP. Combining hypoxic methods for peak performance. Sports Med. 2010;40(1):1–25. 10.2165/11317920-000000000-00000. 20020784 10.2165/11317920-000000000-00000

[198] González-Alonso J, Crandall CG, Johnson JM. The cardiovascular challenge of exercising in the heat. J Physiol. 2008;586(1):45–53. 10.1113/jphysiol.2007.142158. 17855754 10.1113/jphysiol.2007.142158 PMC2375553

[199] Rowell LB. Human cardiovascular adjustments to exercise and thermal stress. Physiol Rev. 1974;54(1):75–159. 10.1152/physrev.1974.54.1.75. 4587247 10.1152/physrev.1974.54.1.75

[200] Périard JD, Racinais S, Sawka MN. Adaptations and mechanisms of human heat acclimation: applications for competitive athletes and sports. Scand J Med Sci Sports. 2015;25(Suppl 1):20–38. 10.1111/sms.12408. 25943654 10.1111/sms.12408

[201] Sawka MN, Leon LR, Montain SJ, Sonna LA. Integrated physiological mechanisms of exercise performance, adaptation, and maladaptation to heat stress. Compr Physiol. 2011;1(4):1883–928. 10.1002/cphy.c100082. 23733692 10.1002/cphy.c100082

[202] Racinais S, Alonso JM, Coutts AJ, et al. Consensus recommendations on training and competing in the heat. Br J Sports Med. 2015;49(18):1164–73. 10.1136/bjsports-2015-094915. 26069301 10.1136/bjsports-2015-094915 PMC4602249

[203] Logan-Sprenger HM, Heigenhauser GJF, Killian KJ, Spriet LL. Effects of dehydration during cycling on skeletal muscle metabolism in females. Med Sci Sports Exerc. 2012;44(10):1949–57. 10.1249/MSS.0b013e31825abc7c. 22543739 10.1249/MSS.0b013e31825abc7c

[204] Logan-Sprenger HM, Heigenhauser GJF, Jones GL, Spriet LL. Increase in skeletal-muscle glycogenolysis and perceived exertion with progressive dehydration during cycling in hydrated men. Int J Sport Nutr Exerc Metab. 2013;23(3):220–9. 10.1123/ijsnem.23.3.220. 23114793 10.1123/ijsnem.23.3.220

[205] Logan-Sprenger HM, Heigenhauser GJF, Jones GL, Spriet LL. The effect of dehydration on muscle metabolism and time trial performance during prolonged cycling in males. Physiol Rep. 2015;3(8):e12483. 10.14814/phy2.12483. 26296770 10.14814/phy2.12483 PMC4562569

[206] Murphy PJ, Campbell SS. Nighttime drop in body temperature: a physiological trigger for sleep onset? Sleep. 1997;20(7):505–11. 10.1093/sleep/20.7.505. 9322266 10.1093/sleep/20.7.505

[207] Okamoto-Mizuno K, Mizuno K. Effects of thermal environment on sleep and circadian rhythm. J Physiol Anthropol. 2012;31(1):14. 10.1186/1880-6805-31-14. 22738673 10.1186/1880-6805-31-14 PMC3427038

[208] Nybo L, Rasmussen P, Sawka MN. Performance in the heat-physiological factors of importance for hyperthermia-induced fatigue. Compr Physiol. 2014;4(2):657–89. 10.1002/cphy.c130012. 24715563 10.1002/cphy.c130012

[209] Zurawlew MJ, Walsh NP, Fortes MB, Potter C. Post-exercise hot water immersion induces heat acclimation and improves endurance exercise performance in the heat. Scand J Med Sci Sports. 2016;26(7):745–54. 10.1111/sms.12638. 26661992 10.1111/sms.12638

[210] Daanen HAM, Racinais S, Périard JD. Heat acclimation decay and re-induction: a systematic review and meta-analysis. Sports Med. 2018;48(2):409–30. 10.1007/s40279-017-0808-x. 29129022 10.1007/s40279-017-0808-x PMC5775394

[211] Casa DJ, Armstrong LE, Hillman SK, et al. National athletic trainers’ association position statement: fluid replacement for athletes. J Athl Train. 2000;35(2):212–24. 16558633 PMC1323420

[212] Shirreffs SM, Maughan RJ. Volume repletion after exercise-induced volume depletion in humans: replacement of water and sodium losses. Am J Physiol. 1998;274(5):F868–75. 10.1152/ajprenal.1998.274.5.F868. 9612323 10.1152/ajprenal.1998.274.5.F868

[213] Bongers CCWG, Thijssen DHJ, Veltmeijer MTW, Hopman MTE, Eijsvogels TMH. Precooling and percooling (cooling during exercise) both improve performance in the heat: a meta-analytical review. Br J Sports Med. 2015;49(6):377–84. 10.1136/bjsports-2013-092928. 24747298 10.1136/bjsports-2013-092928

[214] Tyler CJ, Sunderland C, Cheung SS. The effect of cooling prior to and during exercise on exercise performance and capacity in the heat: a meta-analysis. Br J Sports Med. 2015;49(1):7–13. 10.1136/bjsports-2012-091739. 23945034 10.1136/bjsports-2012-091739

[215] Burgess HJ, Crowley SJ, Gazda CJ, Fogg LF, Eastman CI. Preflight adjustment to eastward travel: 3 days of advancing sleep with and without morning bright light. J Biol Rhythms. 2003;18(4):318–28. 10.1177/0748730403253585. 12932084 10.1177/0748730403253585 PMC1262683

[216] Caballero-Gomez H, Johnston J, Jackson CL, Romano L, Cushing LJ. Ambient and bedroom heat in relation to sleep health in a marginalized community that is one of the hottest in Los Angeles. Int J Environ Res Public Health. 2025;22(9):1391. 10.3390/ijerph22091391. 41007535 10.3390/ijerph22091391 PMC12469264

[217] Cain T, Brinsley J, Bennett H, Nelson M, Maher C, Singh B. Effects of cold-water immersion on health and wellbeing: a systematic review and meta-analysis. PLoS ONE. 2025;20(1):e0317615. 10.1371/journal.pone.0317615. 39879231 10.1371/journal.pone.0317615 PMC11778651

[218] Chevance G, Minor K, Vielma C, et al. A systematic review of ambient heat and sleep in a warming climate. Sleep Med Rev. 2024;75(101915):101915. 10.1016/j.smrv.2024.101915. 38598988 10.1016/j.smrv.2024.101915

[219] Li A, Luo H, Zhu Y, et al. Climate warming may undermine sleep duration and quality in repeated-measure study of 23 million records. Nat Commun. 2025;16(1):2609. 10.1038/s41467-025-57781-y. 40097430 10.1038/s41467-025-57781-y PMC11914098

[220] Vitale KC, Owens R, Hopkins SR, Malhotra A. Sleep hygiene for optimizing recovery in athletes: review and recommendations. Int J Sports Med. 2019;40(8):535–43. 10.1055/a-0905-3103. 31288293 10.1055/a-0905-3103 PMC6988893

[221] Duffield R, Coutts A, McCall A, Burgess D. Pre‐cooling for football training and competition in hot and humid conditions. EJSS (Champaign). 2013;13(1):58–67. 10.1080/17461391.2011.589474.

[222] Stevens CJ, Best R. Menthol: a fresh ergogenic aid for athletic performance. Sports Med. 2017;47(6):1035–42. 10.1007/s40279-016-0652-4. 27858306 10.1007/s40279-016-0652-4

[223] Lee JKW, Shirreffs SM, Maughan RJ. Cold drink ingestion improves exercise endurance capacity in the heat. Med Sci Sports Exerc. 2008;40(9):1637–44. 10.1249/MSS.0b013e318178465d. 18685527 10.1249/MSS.0b013e318178465d

[224] Siegel R, Maté J, Brearley MB, Watson G, Nosaka K, Laursen PB. Ice slurry ingestion increases core temperature capacity and running time in the heat. Med Sci Sports Exerc. 2010;42(4):717–25. 10.1249/MSS.0b013e3181bf257a. 19952832 10.1249/MSS.0b013e3181bf257a

[225] Hawke KV, Gavel EH, Bentley DJ, Logan-Sprenger HM. Menthol mouth rinsing improves cycling performance in trained adolescent males under heat stress. Int J Sport Nutr Exerc Metab. 2025;35(1):34–42. 10.1123/ijsnem.2024-0028. 39214518 10.1123/ijsnem.2024-0028

[226] Gavel EH, Barreto G, Hawke KV, et al. How Cool ? Effects menthol mouth rinsing exercise capacity performance: systematic review meta-analysis 10.1186/s40798-024-00679-8 PMC10881929 38381237

[227] Poppendieck W, Faude O, Wegmann M, Meyer T. Cooling and performance recovery of trained athletes: a meta-analytical review. Int J Sports Physiol Perform. 2013;8(3):227–42. 10.1123/ijspp.8.3.227. 23434565 10.1123/ijspp.8.3.227

